# Supplementary material for: Streptomyces alleviate abiotic stress in plant by producing pteridic acids
Source: Nat Commun. 2023 Nov 15;14:7398. doi: 10.1038/s41467-023-43177-3 (PMC10652019; doi:10.1038/s41467-023-43177-3)
Supplement: Supplementary file 1 — Supplementary Information [file 41467_2023_43177_MOESM1_ESM.pdf]

Supplementary Information

Streptomyces alleviate abiotic stress in plant by producing pteridic acids

Zhijie Yang[1], Yijun Qiao[1], Naga Charan Konakalla[2], Emil Strøbech[1], Pernille Harris[3], Gundela Peschel[4], Miriam Agler-Rosenbaum[4], Tilmann Weber[5], Erik Andreasson[2], Ling Ding[1,*]

[1] Department of Biotechnology and Biomedicine, Technical University of Denmark, Søltofts Plads, Building 221, 2800 Kgs. Lyngby, Denmark.

[2] Department of Plant Protection Biology, Swedish University of Agricultural Sciences, Sundsvägen 14, SE-230 53, Alnarp, Sweden.

[3] Department of Chemistry, Technical University of Denmark, Søltofts Plads, Building 206, 2800 Kgs. Lyngby, Denmark.

[4] Leibniz Institute for Natural Product Research and Infection Biology - Hans Knöll Institute (HKI), Beutenbergstr. 11a, 07745 Jena, Germany.

[5] Novo Nordisk Foundation Center for Biosustainability, Technical University of Denmark, Kemitorvet, Building 220, 2800 Kgs. Lyngby, Denmark.

*Corresponding author: Ling Ding lidi@dtu.dk

Table of Contents

Content	Page
**Tab. 1**. The biosynthetic gene clusters in *S. iranensis*.	4
**Tab. 2**. The annotation of pteridic acids core biosynthetic genes in *S. iranensis*.	6
**Tab. 3**. Alignment of conserved motifs in the active site of AT domains.	7
**Tab. 4**. The list of *Streptomyces* strains that can produce pteridic acids, elaiophylin or harboring *pta* gene cluster.	8
**Tab. 5**. Summary of strains and plasmids used in this study.	10
**Tab. 6**. ^{1}H (800 MHz) NMR data for **1–2** (in MeOD).	11
**Tab. 7**. ^{13}C (200 MHz) NMR data for **1–2** (in MeOD).	12
**Tab. 8**. Summary of primers used in this study.	13
**Fig. 1**. Enrichment evaluation of *S. iranensis* in rhizosphere soil.	14
**Fig. 2**. Barley experiments of *S. iranensis* and *S. iranensis*/Δ*ptaA* treatment.	14
**Fig. 3**. Mirror plots comparing spectra from known metabolites from *S. iranensis* to standard spectra deposited in GNPS.	15
**Fig. 4**. ^{1}H NMR spectrum of **1.**	16
**Fig. 5**. ^{13}C NMR spectrum of **1.**	16
**Fig. 6**. NOESY spectrum of **1**.	17
**Fig. 7**. COSY spectrum of **1**.	17
**Fig. 8**. HSQC spectrum of **1**.	18
**Fig. 9**. HMBC spectrum of **1**.	18
**Fig. 10**. Mass spectrum of **1**.	19
**Fig. 11**. Selected HMBC correlations for **1** and **2**.	19
**Fig. 12**. Crystal structure of **1**.	19
**Fig. 13**. ^{1}H NMR spectrum of **2**.	20
**Fig. 14**. ^{13}C NMR spectrum of **2**.	20
**Fig. 15**. COSY spectrum of **2**.	21
**Fig. 16**. NOESY spectrum of **2**.	21
**Fig. 17**. H2BC spectrum of compound **2**.	22
**Fig. 18**. HSQC spectrum of **2**.	22
**Fig. 19**. HMBC spectrum of **2**.	23
**Fig. 20**. Mass spectrum of **2**.	23
**Fig. 21**. Primary root length of *Arabidopsis* seedling treated with different concentrations of pteridic acids H and F.	24
**Fig. 22**. Inter-sample correlation heat map.	24
**Fig. 23**. Kidney beans growth experiment with pure pteridic acids.	25
**Fig. 24**. Pteridic acid H and ABA at 1 ng mL^{-1} help Mung beans against heavy metal stress.	25
**Fig. 25**. Multiple sequence alignment of KR domains.	26
**Fig. 26**. Multiple sequence alignment of DH domains.	27
**Fig. 27**. Stability test of pteridic acids.	28
**Fig. 28**. The schematic of optimized genetic manipulation in *S. iranensis* by using CRISPR-cBEST system.	29
**Fig. 29**. Complementation experiment of *ptaA*-inactivation mutant of *S. iranensis*.	30
**Fig. 30**. Multiple sequence alignment of TE domains.	31
**Fig. 31**. The HR-LC-MS analysis of pteridic acids in *S. violaceusniger* Tu 4113 and *S. rapamycinicus* NRRL 5491.	32
**Fig. 32.** Abiotic stresses alleviation led by *S. violaceusniger* Tu 4113 and *S. rapamycinicu*s NRRL 5491	33
**Fig. 33**. The phylogenetic analysis of potential pteridic acids *Streptomyces* producers.	34
**Fig. 34**. Genome similarity analysis based on the alignment of 15 sequenced *pta*-containing *Streptomyces* genomes.	35
**Fig. 35**. The genome synteny analysis of 15 *pta*-containing *Streptomyces* strains.	36
**Fig. 36**. Identification of pteridic acids and elaiophylin in *S. albus* DSM 41398.	37
**Fig. 37**. The secondary metabolites biosynthetic gene clusters profiles of 15 *pta*-containing *Streptomyces* strains with complete genome information.	38

**Fig. 38**. The secondary metabolites BGCs similarity network of 15 pta-containing *Streptomyces* strains with complete genome information.	38
**References**	39

Tab. 1. The predicted biosynthetic gene clusters in *S. iranensis*.

Cluster	Type	Position	Most similar known cluster	Similarity
Cluster 1	T1PKS,transAT-PKS	49–114,601	sceliphrolactam	32%
Cluster 2	terpene,NRPS	161,367-220,243	carotenoid	63%
Cluster 3	NRPS	436,587-485,770	coelichelin	100%
Cluster 4	butyrolactone	773,681-782,636	cyphomycin	9%
Cluster 5	phosphonate,acyl_amino_acids, butyrolactone,NRPS-like, T1PKS,hserlactone	837,187-987,392	chlorothricin / deschlorothricin	13%
Cluster 6	T1PKS	1,074,827-1,216,558	azalomycin F3a	100%
Cluster 7	T1PKS	1,388,946-1,573,704	nigericin	100%
Cluster 8	T1PKS	1,677,611-1,756,225	elaiophylin	87%
Cluster 9	redox-cofactor	1,780,894-1,802,958		
Cluster 10	hserlactone	1,920,187-1,940,942	daptomycin	4%
Cluster 11	butyrolactone	1,951,656-1,962,588		
Cluster 12	T1PKS,NRPS	2,141,406-2,193,626	meilingmycin	4%
Cluster 13	NRPS,T3PKS,other	2,252,019-2,351,341	feglymycin	78%
Cluster 14	NRPS,T1PKS	2,405,901-2,618,214	pentamycin	46%
Cluster 15	terpene	2,865,078-2,887,266	hopene	76%
Cluster 16	T1PKS	3,033,413-3,115,318	geldanamycin	34%
Cluster 17	T2PKS	3,184,430-3,254,311	spore pigment	83%
Cluster 18	T1PKS	3,378,842-3,439,037	s56-p1	11%
Cluster 19	RiPP-like	3,492,148-3,502,022		
Cluster 20	siderophore	3,672,342-3,682,811		
Cluster 21	T2PKS	4,017,972-4,090,475	isoindolinomycin	61%
Cluster 22	NRPS-like	4,320,588-4,352,287	echoside A / echoside B / echoside C / echoside D / echoside E	100%
Cluster 23	siderophore	5,114,280-5,125,349	desferrioxamin B	100%
Cluster 24	terpene	6,324,614-6,344,943	geosmin	100%
Cluster 25	ladderane,arylpolyene,NRPS	6,588,275-6,690,850	RP-1776	48%
Cluster 26	NRPS	7,228,322-7,270,720	ochronotic pigment	75%
Cluster 27	Ladderane	7,332,623-7,372,653	atratumycin	34%
Cluster 28	RRE-containing	7,391,754-7,414,213	granaticin	10%
Cluster 29	T1PKS	7,547,548-7,732,661	mediomycin A	68%
Cluster 30	terpene	8,282,077-8,302,096		
Cluster 31	ectoine	8,948,675-8,959,079	ectoine	100%
Cluster 32	siderophore	9,122,543-9,136,295		
Cluster 33	terpene	9,187,376-9,204,614	BE-43547A1 / BE-43547A2 / BE-43547B1 / BE-43547B2 / BE-43547B3 / BE-43547C1 / BE-43547C2	25%
Cluster 34	nucleoside	9,422,403-9,459,458	toyocamycin	40%
Cluster 35	nucleoside,NRPS,T1PKS,NRPS-like	9,564,087-9,711,679	rapamycin	82%
Cluster 36	PKS-like	9,800,077-9,841,105	rustmicin	33%
Cluster 37	terpene	9,891,287-9,911,108	2-methylisoborneol	100%
Cluster 38	terpene	10,356,847-10,375,970	pristinol	100%
Cluster 39	T1PKS,ladderane,arylpolyene	10,470,251-10,521,311	atratumycin	28%

Cluster 40	NRPS-like,T1PKS,transAT-PKS	10,727,870-10,814,731	hygrocin A / hygrocin B	83%
Cluster 41	lanthipeptide-class-ii	10,908,405-10,931,776	reveromycin A	9%
Cluster 42	hglE-KS,T1PKS,RiPP-like	10,960,035-11,021,175		
Cluster 43	T1PKS,NRPS	11,024,823-11,167,452	ambruticin	17%
Cluster 44	terpene	11,218,988-11,239,914	brasilicardin A	30%
Cluster 45	terpene	11,368,510-11,389,418		
Cluster 46	NRPS-like,T1PKS	11,474,253-11,522,775	niphimycins C-E	9%
Cluster 47	betalactone	11,718,451-11,747,262	Sch-47554 / Sch-47555	7%

Tab. 2. The annotation of pteridic acids core biosynthetic genes in *S. iranensis*.

ORF	Size[a]	Proposed functions	SI/ID[b]	Protein homologue and origin
Pta5	1041	3-oxoacyl-ACP synthase III	98/97	WP_210951575.1, *Streptomyces* sp. MK37H
pta4	572	3-hydroxyacyl-CoA dehydrogenase	98/97	WP_020866330.1, *Streptomyces rapamycinicus*
pta3	958	LuxR family transcriptional regulator	99/99	WP_020866329.1, *Streptomyces rapamycinicus*
pta2	316	glucose-1-phosphate thymidylyltransferase	99/99	RLV74943.1, *Streptomyces rapamycinicus* NRRL 5491
pta1	324	TDP glucose 4,6 dehydratase	98/98	WP_214665150.1, *Streptomyces javensis*
ptaA	4535	Type I PKS	90/88	WP_214609358.1, *Streptomyces malaysiensis*
ptaB	1746	Type I PKS	91/89	WP_037957959.1, *Streptomyces* sp. PRh5
ptaC	1655	Type I PKS	95/94	WP_138910801.1, *Streptomyces* sp. DASNCL29
ptaD	3395	Type I PKS	96/95	WP_020866322.1, Streptomyces rapamycinicus
ptaE	2112	Type I PKS	95/95	WP_201848053.1, *Streptomyces* sp. 110
*pta1**	261	Thioesterase	97/95	GDY58888.1, *Streptomyces violaceusniger*
*pta2**	417	Glycosyltransferase	99/98	MBP8534388.1, *Streptomyces* sp. MK37H
*pta3**	196	dTDP-4-dehydrorhamnose 3,5-epimerase	97/97	WP_210946413.1, *Streptomyces* sp. MK37H
*pta4**	304	Exporter (membrane domain)	100/99	WP_138910796.1, *Streptomyces* sp. DASNCL29
*pta5**	245	Exporter (ATPase domain)	99/99	WP_020866316.1, *Streptomyces rapamycinicus*
*pta6**	420	Two-component regulator (sensor/kinase domain)	97/97	WP_199334865.1, *Streptomyces* sp. GMR22
*pta7**	223	Two-component regulator (effector domain)	98/98	WP_138910795.1, *Streptomyces* sp. DASNCL29
*pta8**	321	NAD(P)-dependent oxidoreductase	96/96	WP_164428021.1, *Streptomyces rhizosphaericus*
*pta9**	328	aldo/keto reductase	98/98	WP_191066610.1, *Streptomyces* sp. 5-10
*pta10**	469	NDP-hexose 2,3 dehydratase	97/95	GDY58900.1, *Streptomyces violaceusniger*
*pta11**	446	Crotonyl-CoA reductase	99/99	WP_020866310.1, *Streptomyces rapamycinicus*

a, The size of amino acids; b, similarity-identity ratio.

Tab. 3. Alignment of conserved motifs in the active site of AT domains.

**domain name**	**specificity**	**198**	**199**	**200**	**201**
ave_AT5	Malonyl-CoA	H	A	F	H
nid_AT3	Malonyl-CoA	H	A	F	H
epo_AT2	Malonyl-CoA	H	A	F	H
amp_AT18	Malonyl-CoA	H	A	F	H
rif_AT2	Malonyl-CoA	H	A	F	H
pta_LD	Malonyl-CoA	H	A	F	H
pta_AT2	Malonyl-CoA	H	A	F	H
pta_AT6	Malonyl-CoA	H	A	F	H
pta_AT7	Malonyl-CoA	I	A	A	H
ave_AT1	Methylmalony-CoA	Y	A	S	H
nid_AT4	Methylmalony-CoA	Y	A	S	H
ery_AT4	Methylmalony-CoA	Y	A	S	H
amp_AT2	Methylmalony-CoA	Y	A	S	H
rif_AT7	Methylmalony-CoA	Y	A	S	H
pta_AT3	Methylmalony-CoA	Y	A	S	H
pta_AT4	Methylmalony-CoA	Y	A	S	H
pta_AT5	Methylmalony-CoA	Y	A	S	H
nid_AT5	Ethylmalonyl-CoA	T	A	G	H
tyl_AT5	Ethylmalonyl-CoA	T	A	G	H
pta_AT1	Ethylmalonyl-CoA	T	A	G	H
ery_LD	Propionyl-CoA	M	A	A	H
meg_LD	Propionyl-CoA	M	A	A	H
epo_AT3	Flexible	H	A	S	H

* Abbreviations: LD, loading module; ave, avermectin; nid, niddamycin; epo, epothilone; amp, amphotericin; rif, rifamycin; ery, erythromycin; tyl, tylactone; meg, megalomicin; pta, pteridic acids.

Tab. 4. The *Streptomyces* strains that can produce elaiophylin, pteridic acids, or harbor *pta* BGC.

NO	Strain name	Geographic location	Source	Reference/Assembly
1	*Streptomyces iranensis* HM 35	Isfahan City, Iran	rhizosphere	GCA_000938975.1
2	*Streptomyces* sp. 219807	Sanya, Hainan, China	mangrove soil	1
3	*Streptomyces* sp. SCSGAA 0027	South China Sea, China	gorgonian-associated	2
4	*Streptomyces* sp. 7-145	Heishijiao Bay, Dalian, China	marine-sediment	3
5	*Streptomyces melanosporofaciens*	Italy	soil	4
6	*Streptomyces autolyticus* CGMCC 0516	Yunnan, China	soil	5
7	*Streptomyces* sp. DSM 3816	Kypcerissia, Greece	soil	6
8	*Streptomyces* sp. BCC 71188	Nakhon Si Thammarat Province, Thailand	soil	7
9	*Streptomyces* sp. BCC 72023	Chumphon province, Thailand	plant-associated	8
10	*Streptomyces* sp. BS 1261	New Zealand	soil	9
11	*Streptomyces* sp. ICBB 9297	Jatiroto, East Java, Indonesia	soil	10
12	*Streptomyces* sp. SNA-4606	Towada-shi, Aomori Prefecture, Japan	soil	11
13	*Streptomyces* sp. MCY-846	cheju-island, Korea	soil	12
14	*Streptomyees albiflaviniger* SCSIO ZJ28	South China Sea, China	marine-sediment	13
15	*Streptomyces* sp. USC-16018	Hastings Point, NSW, Australia	marine	14
16	*Streptomyces* sp. SPMA113	Prajinburi Province, Thailand	soil	15
17	*Streptomyces* sp. LZ35	Ji'mei, Xia'men, China	soil	16
18	*Streptomyces malaysiensis* DSM 4137	Germany	soil	17
19	*Streptomyces* sp. BCa1	Borra Caves, India	soil	18
20	*Streptomyces* sp. IFM11958	Sakuragi cemetery, Chiba city, Japan	soil	19
21	*Streptomyces malaysiensis* OUCMDZ-2167	South China Sea, China	marine	20
22	*Streptomyces* sp. 11-1-2	Newfoundland, Canada	plant-associated	21
23	*Streptomyces* sp. HNM0561	Hainan, China	marine-sediment	22
24	*Streptomyces* sp. GMR 22	Wanagama Forest, Indonesia	soil	23
25	*Streptomyces* sp. NTK 935	Canary Basin	marine sediment	24
26	*Streptomyces* sp. NTK 937	Canary Basin	marine sediment	24
27	*Streptomyces* sp. RJA 2928	Papua New Guinea	marine sediment	25
28	*Streptomyces rapamycinicus* NRRL 5491	Easter island, Fiji	soil	26
29	*Streptomyces pseudouerticillus* YN 17707	Xishuangbanna, Yunnan, China	soil	27
30	*Streptomyces* sp. SCSIO ZS0520	Okinawa, Japan	marine sediment	28
31	*Streptomyces hygroscopicus* NND-52	Suqian, Jiangsu, China	soil	29
32	*Streptomyces* hygroscopicus NO.662	Sapporo-city, Hokkaido, Japan	soil	30
33	*Streptomyces hygroscopicus* TP-A0451	Toyama, Japan	plant-associated	31
34	*Streptomyces hygroscopicus* CH-7	Vojvodina, Serbia	soil	32
35	*Streptomyces hygroscopicus* 17997	Yunnan, China	soil	33
36	*Streptomyces hygroscopicus* ACTMS-9H	Amazon, Brazil	rhizosphere	34
37	*Streptomyces hygroscopicus* XM 201	Xiamen, Fujian, China	soil	35
38	*Streptomyces yatensis* DSM 41771	New Caledonia	Ultramafic soil	36
39	*Streptomyces solisilvae* HNM0141	Bawangling, Hainan, China	soil	37
40	*Streptomyces* sp. NA02950	Hainan, China	marine-sediment	38

41	*Streptomyces* sp. PRh5	Dongxiang, China	plant-associated	39
42	*Streptomyces albus* DSM 41398	Fuji City, Shizuoka Pref, Japan	soil	GCA_000827005.1
43	*Streptomyces samsunensis* SA31	Songkhla, Thailand	soil	GCA_013345665.1
44	*Streptomyces* sp. MK37H	Antalya, Turkey	soil	GCA_018035285.1
45	*Streptomyces* sp. 4503	Guangxi, China	mangrove sediment	GCA_018883605.1
46	*Streptomyces* sp. t39	Austin, Texas, USA	soil	GCA_008042045.1
47	*Streptomyces* sp. 5-10	Hainan, China	plant-associated	GCA_014712245.1
48	*Streptomyces antioxidans* MUSC164	Malaysia	mangrove	GCA_000968685.2
49	*Streptomyces malaysiensis* F913	Chongqing, China	soil	GCA_002891865.1
50	*Streptomyces* sp. DASNCL29	Unkeshwar, India	soil	GCA_005938145.1
51	*Streptomyces* sp. WAC05858	Germeny	soil	GCA_003949695.1
52	*Streptomyces rhizosphaericus* 0250	Taian, China	soil	GCA_010892295.1
53	*Streptomyces* sp. NEAU-YJ-81	Harbin, China	soil	GCA_017592595.1
54	*Streptomyces rhizosphaericus* NRRL B-24304	Indonesia	rhizosphere	GCA_002155885.1
55	*Streptomyces malaysiensis* TY049-057	Bidor Perak, Malaysia	soil	GCA_008033485.1
56	*Streptomyces malaysiensis* DSM 14702	Germany	soil	GCA_011800555.1
57	*Streptomyces cangkringensis* DSM 41769	South Korea	-	GCA_019059395.1
58	*Streptomyces endocoffeicus* CA3R110	Lampang, Thailand	plant-associated	GCA_016741935.1
59	*Streptomyces indonesiensis* DSM 41759	Yogyakarta, Indonesia	rhizosphere	GCA_018138705.1
60	*Streptomyces rhizosphaericus* DSM 41760	Yogyakarta, Indonesia	rhizosphere	GCA_017942185.1
61	*Streptomyces asiaticus* DSM 41761	Yogyakarta, Indonesia	rhizosphere	GCA_018138715.1
62	*Streptomyces* sp. RCU064	Nong Jum Rung, Thailand	Peat swamp forest soil	GCA_024505145.1
63	*Streptomyces violaceusniger* Tu 4113	-	soil	GCA_000147815.3
64	*Streptomyces antimycoticus* NBRC 100767	-	soil	GCA_009936315.1
65	*Streptomyces antimycoticus* NBRC 12839	-	soil	GCA_005405925.1
66	*Streptomyces* sp. AgN23	-	rhizosphere	GCA_001598115.2
67	*Streptomyces* sp. NRRL 30748	-	soil	39
68	*Streptomyces* sp. M56	-	termite-associated	40
69	*Streptomyces* sp. CWJ-256	-	plant-associated	41
70	*Streptomyces* sp. 92JF-1	-	marine	42
71	*Streptomyces* sp. KIB-H869	-	plant-associated	43
72	*Streptomyces hygroscopicus* MSU-625	-	soil	44
73	*Streptomyces hygroscopicus* MSU-616	-	soil	45
74	*Streptomyces hygroscopicus* OUPS-N92	-	marine	46
75	*Streptomyces* sp. CBR53	-	-	47
76	*Streptomyces violaceusniger* NBRC 13459	-	-	GCA_005405945.1
77	*Streptomyces violaceusniger* NRRL F-8817	-	-	GCA_001509775.1
78	*Streptomyces* sp. HKI-0113	-	-	48
79	*Streptomyces* sp. HKI-0114	-	-	48
80	*Streptomyces* sp. 57-13	-	-	49
81	*Streptomyces javensis*	-	-	GCA_016103505.1

“-”: information missing.

Tab. 5. Summary of strains and plasmids used in this study.

**Strains**	**Description**	**Source/[Ref]**
One Shot™ Mach1™ T1 Phage-Resistant Chemically Competent *E. coli*	For routine plasmids maintenance and cloning	Thermo Fisher Scientific
E. coli ET12567/pUZ8002	For conjugating plasmids into *Streptomyces*	[50]
S. iranensis	Wild-type strain	DSMZ
S. iranensis/Δ*ptaA*	Δ*ptaA* mutant strain	In this work
*S. iranensis/*Δ*ptaA/*1J23	Complementation strain of Δ*ptaA* mutant	In this work
S. iranensis/Δ*ptaA/*6M10	Complementation strain of Δ*ptaA* mutant	In this work
S. iranensis/M2089I + E2090K + D2091N	TE domain mutant strain	In this work
S. rapamycinicus NRRL 5491	Wild-type strain	DSMZ
S. violaceusniger Tu 4113	Wild-type strain	DSMZ
S. albus DSM 41398	Wild-type strain	DSMZ
**Plasmids**		
pCRISPR-cBEST	For C to T base editing	[51]
pCRISPR-cBEST/Δ*ptaA*	Modified plasmid for inactivation of *ptaA*	In this work
pCRISPR-cBEST/ M2089I + E2090K + D2091N	Modified plasmid for site-specific mutation of TE domain	In this work
pESCA13/1J23	BAC for complementation	In this work
pESCA13/6M10	BAC for complementation	In this work

Tab. 6. ^{1}H (800 MHz) NMR data for **1–2** (in MeOD).

position	Δ_H (J in Hz)	
	**1**	**2**
1	-	-
2	5.90 (d, 15.4)	5.97 (d, 15.1)
3	7.33 (dd, 15.4, 11.1)	7.16 (dd, 15.1, 10.9)
4	6.26 (dd, 15.2, 10.8)	6.25 (dd, 15.1, 10.9)
5	6.13 (dd, 15.3, 8.8)	6.07 (dd, 15.1, 8.6)
6	2.48 (m)	2.49 (m)
7	3.85 (dd, 10.2, 2.2)	3.32 (m)
8	2.01 (m)	2.02 (m)
9	3.69 (not determined)	3.56 (dd, 11.5, 4.7)
10	1.61 (m)	1.69 (m)
11	-	-
12	2.29 (dd, 14.9, 6.1), 1.64 (dd, 14.9, 1.9)	2.19 (dd, 13.1, 4.3)<br>1.32 (dd, 13.2, 11.2)
13	3.59 (m)	3.66 (td, 10.8, 4.3)
14	1.49 (m)	1.02 (m)
15	3.43 (m)	3.88 (m)
16	1.21 (d, 6.1)	1.14 (d, 6.2)
17	1.01 (d, 6.8)	1.02 (d, 6.8)
18	0.91 (d, 7.0)	0.95 (d, 6.9)
19	0.96 (d, 6.8)	0.98 (d, 6.8)
20	1.52 (m), 1.21 (m)	1.60 (m), 1.44 (m)
21	0.93 (t, 7.3)	0.82 (t, 7.6)

Tab. 7. ^{13}C (200 MHz) NMR data for **1–2** (in MeOD).

position	*Δ*C, type	
	**1**	**2**
1	168.7	170.2
2	120.6	122.9
3	146.7	148.1
4	129.6	129.6
5	151.0	148.1
6	40.4	40.5
7	75.5	78.0
8	37.5	38.0
9	70.3	74.7
10	42.2	42.1
11	103.2	103.2
12	37.4	33.8
13	70.1	66.3
14	51.3	52.2
15	72.8	66.3
16	20.9	20.4
17	16.0	15.9
18	5.0	5.3
19	12.1	12.7
20	24.8	19.7
21	10.1	10.5

Tab. 8. Summary of primers used in this study.

Primer name	Sequence (5' → 3')	Description
Del-*ptaA*	CGGTTGGTAGGATCGACGGCGCACCCAGGC GGTATGCGTAGTTTTAGAGCTAGAAATAGC	Inactivation of *ptaA*, the base marked in red is sgRNA sequence
Mut-*ptaE*	CCGGTTGGTAGGATCGACGGGGTCCTCCAT CATGGTGAAGGTTTTAGAGCTAGAAATAGC	Site-directed mutagenesis of TE domain in *ptaE*, the base marked in red is sgRNA sequence
ID-sgRNA-F	TGTGTGGAATTGTGAGCGGATA	Forward primer for screening plasmid
ID-sgRNA-R	CCCATTCAAGAACAGCAAGCA	Reverse primer for screening plasmid
ID-*ptaA*-F	TTGCACAGCTCGACGGACAT	Forward primer for screening *ptaA* mutants
ID-*ptaA*-R	GTGTCACCCGCTTTGTCGA	Reverse primer for screening *ptaA* mutants
ID-*ptaE*-F	CAACGCCATGATCGTCGTTC	Forward primer for screening TE domain mutants
ID-*ptaE*-R	CGTTCGAGACCGGGAAATG	Reverse primer for screening TE domain mutants
ID-1J23-right-F	GTCGACATGGCTTGCCTC	Forward primer for validating the right flank of *S. iranensis*/Δ*ptaA*/ 1J23
ID-1J23-right-R	ATCCGTCTCGACTCCGG	Reverse primer for validating the right flank of *S. iranensis*/Δ*ptaA*/ 1J23
ID-1J23-left-F	AGCAGAAGGTAGGGCAG	Forward primer for validating the left flank of *S. iranensis*/Δ*ptaA*/ 1J23
ID-1J23-left-R	GAGGAGACTTCTGCCATGTC	Reverse primer for validating the left flank of *S. iranensis*/Δ*ptaA*/ 1J23
ID-6M10-right-F	GATCTGCTGCTGTTCACGG	Forward primer for validating the right flank of *S. iranensis*/Δ*ptaA*/ 6M10
ID-6M10-right-R	CCGAGCAGATCCGAGATG	Reverse primer for validating the right flank of *S. iranensis*/Δ*ptaA*/ 6M10
ID-6M10-left-F	GAGCACCATCAGCAGGCG	Forward primer for validating the left flank of *S. iranensis*/Δ*ptaA*/ 6M10
ID-6M10-left-R	CATGATGTCCGTGTCGCTC	Reverse primer for validating the left flank of *S. iranensis*/Δ*ptaA*/ 6M10

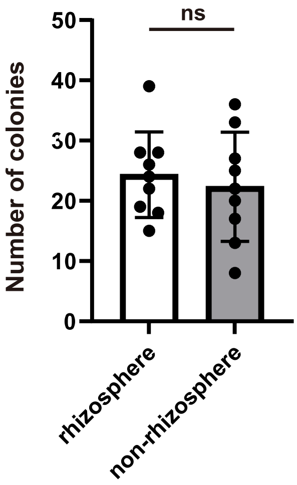

Fig. 1. Enrichment evaluation of *S. iranensis* in rhizosphere soil. Statistical significance was assessed by the unpaired T-test. Source data are provided as a Source Data file.

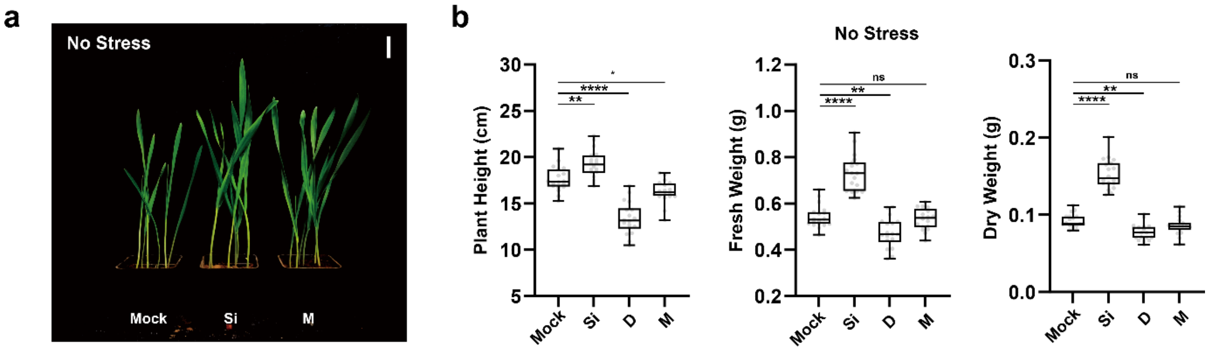

Fig. 2. Barley experiments of *S. iranensis* and *S. iranensis*/Δ*ptaA* treatment under non-stress condition. ***a***, *S. iranensis* and its plant growth-promoting activity on barley seedlings growth (bars=2 cm); ***b***, the box-plots depict the plant height, fresh weight and dry weight of barley seedlings growing on non-stress condition (data is mean ± SD, n =18); Abbreviation: *Mock*, control; *Si*, treatment of *S. iranensis* culture broth; *D*, treatment of *S. iranensis*/Δ*ptaA* culture broth; *M*, treatment of blank medium (ISP2). Statistical significance was assessed by one-way ANOVA with post hoc Dunnett's multiple comparisons test. Asterisks indicate the level of statistical significance: *$p < 0.05$, **$p < 0.01$, ***$p < 0.001$ and ****$p < 0.0001$. All box plots with center lines showing the medians, boxes indicating the interquartile range, and whiskers indicating a range of minimum to maximum data beyond the box. Source data are provided as a Source Data file.

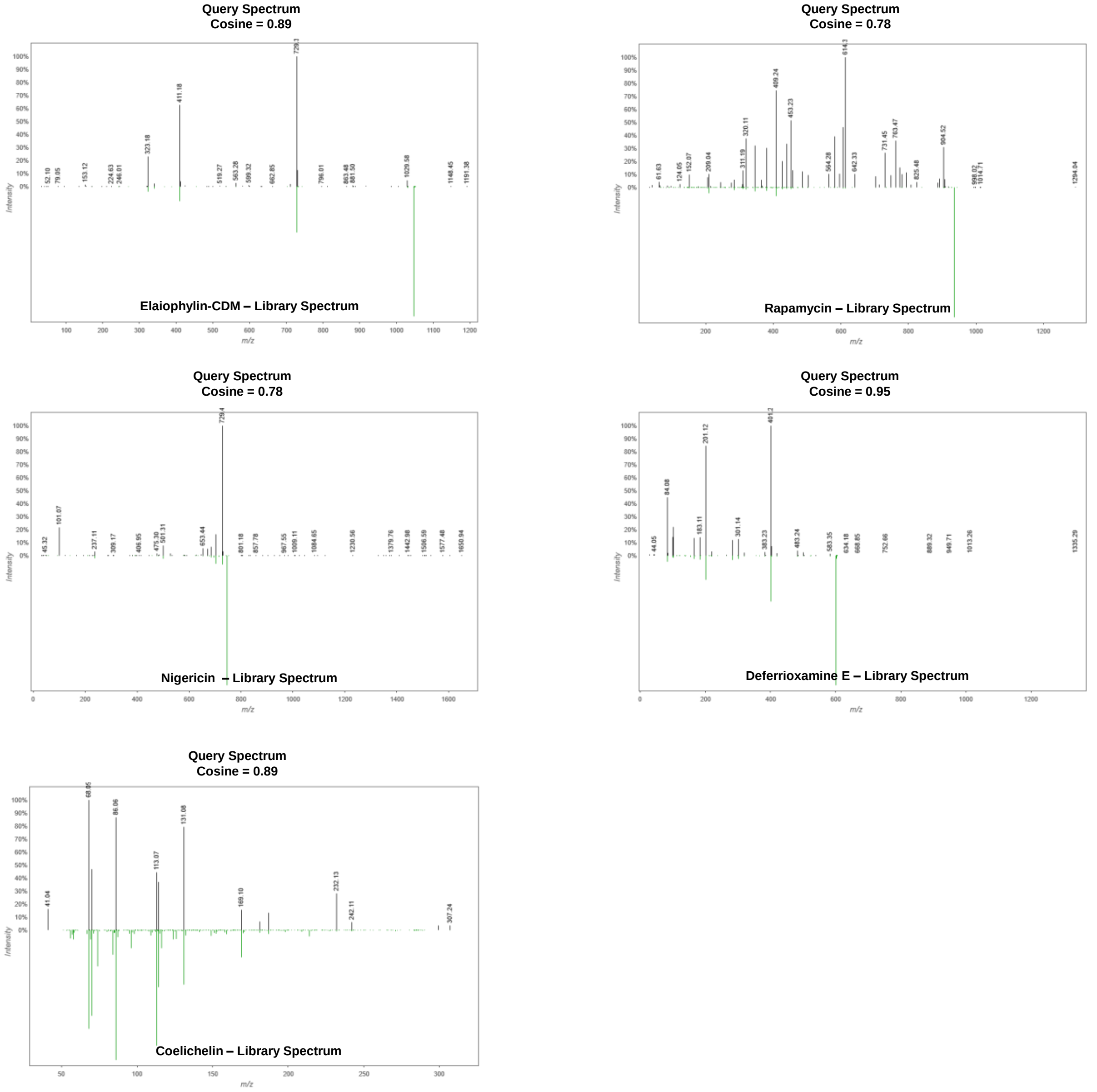

Fig. 3. Mirror plots comparing spectra from known metabolites from *S. iranensis* to standard spectra deposited in GNPS. In the upper part of the plot (black lines) is represented the MS spectra of the candidate feature and in the lower part (green lines) is the MS spectra of the standard compound. Mirror plots have been generated using https://metabolomics-usi.ucsd.edu/.

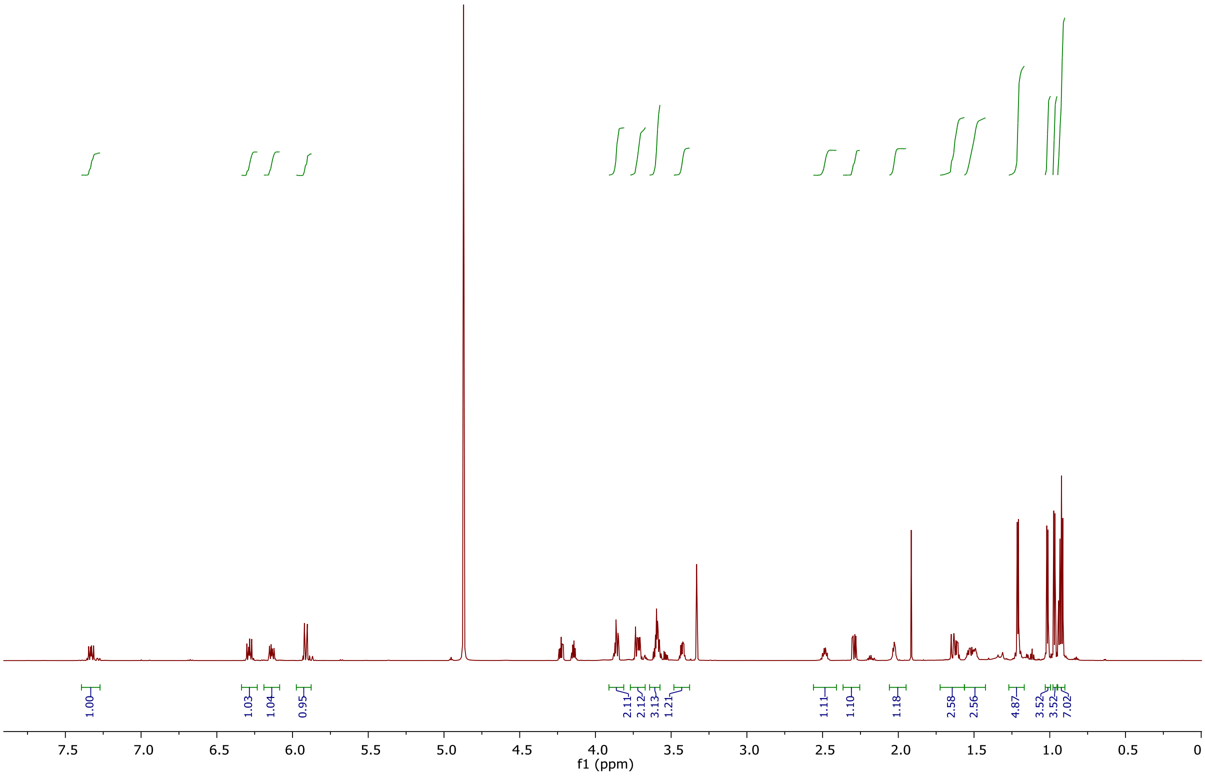

Fig. 4. ^{1}H NMR spectrum of **1.**

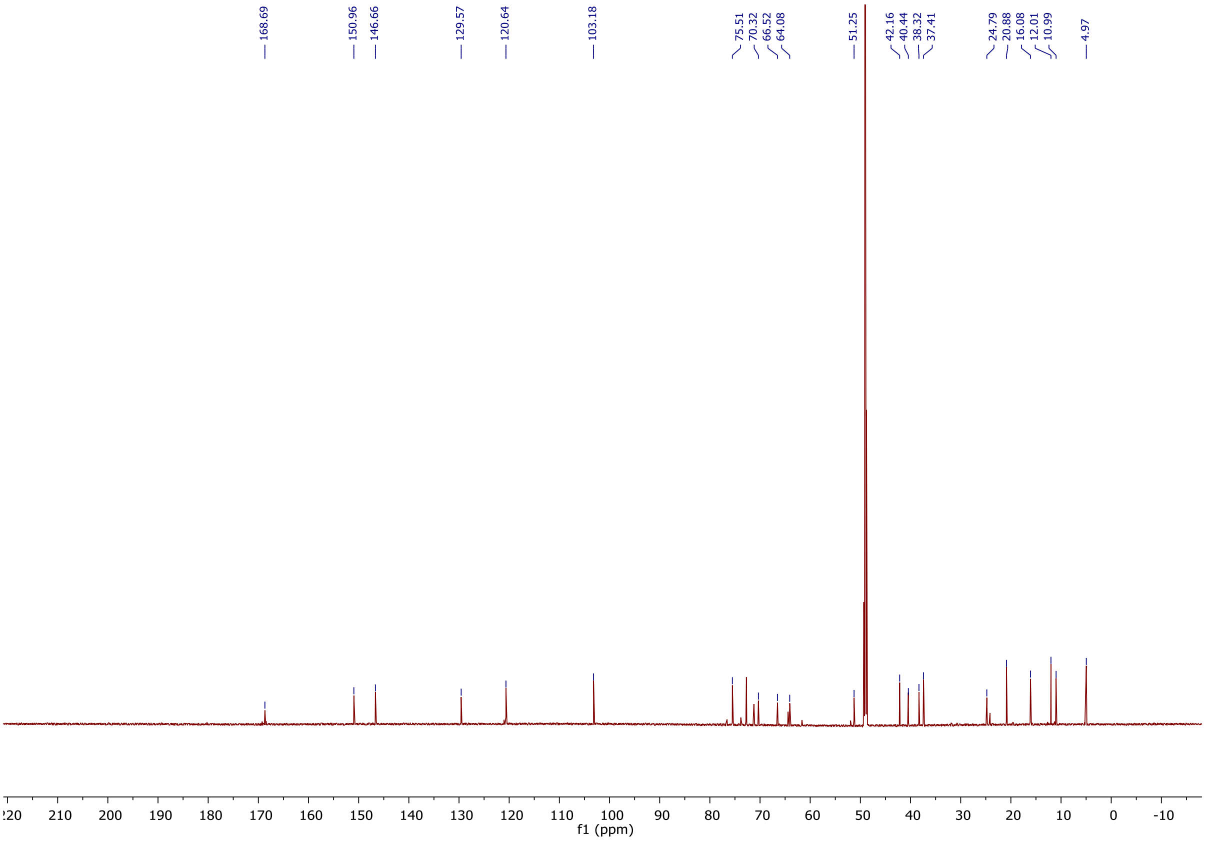

Fig. 5. ^{13}C NMR spectrum of **1.**

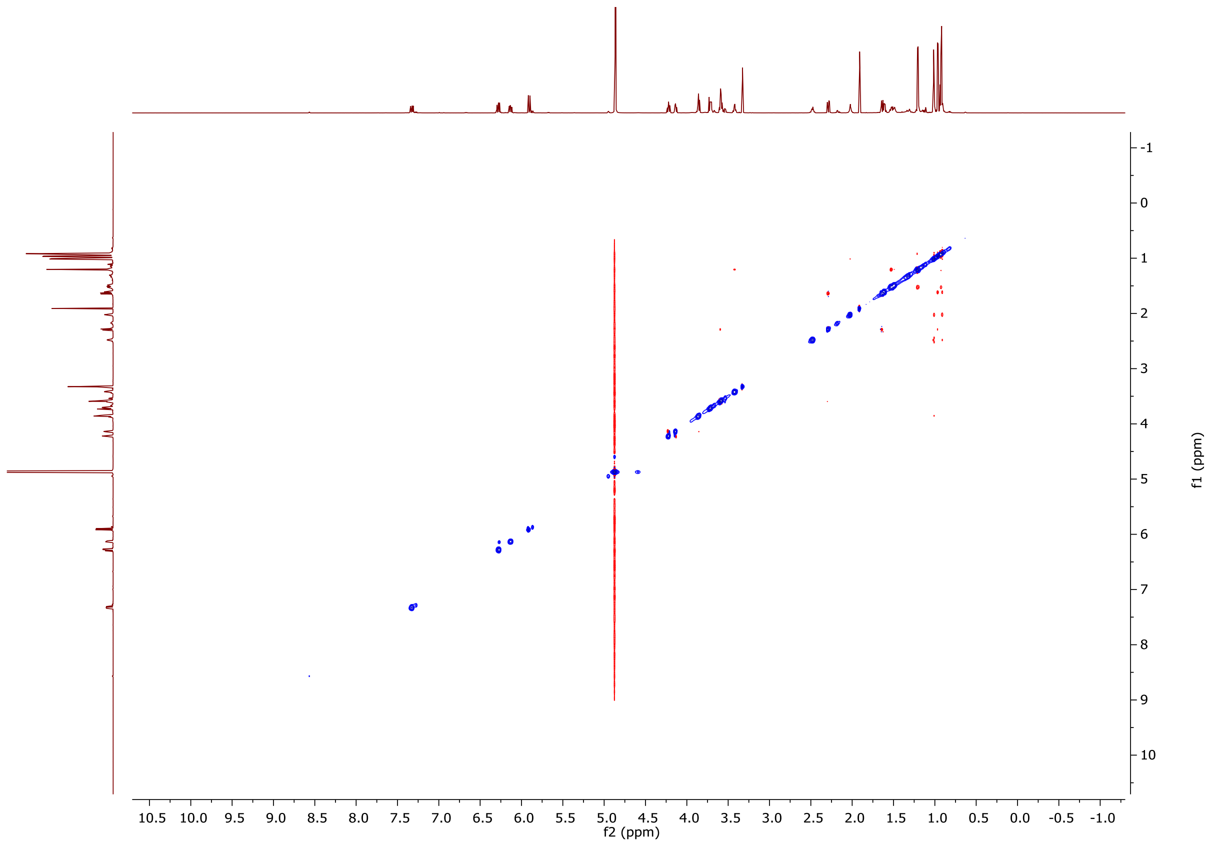

Fig. 6. NOESY spectrum of **1**.

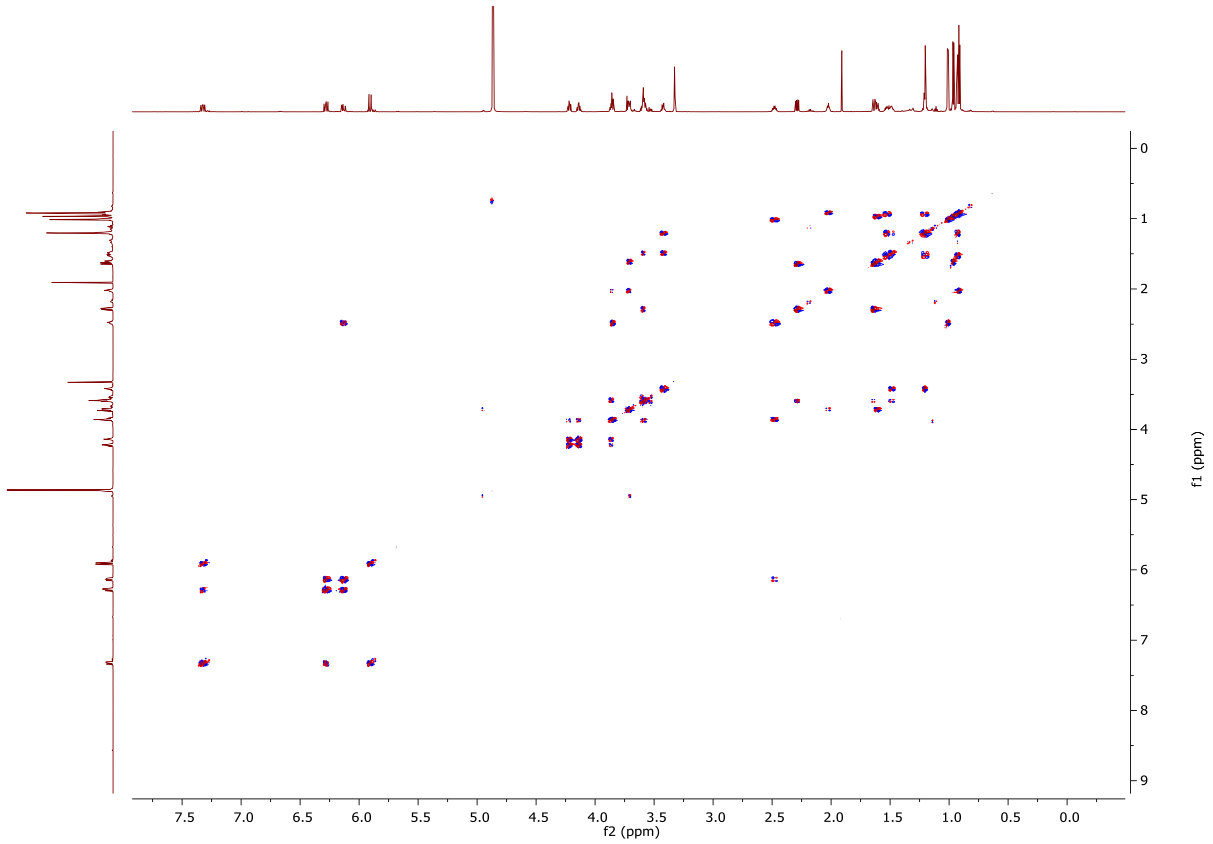

Fig. 7. COSY spectrum of **1**.

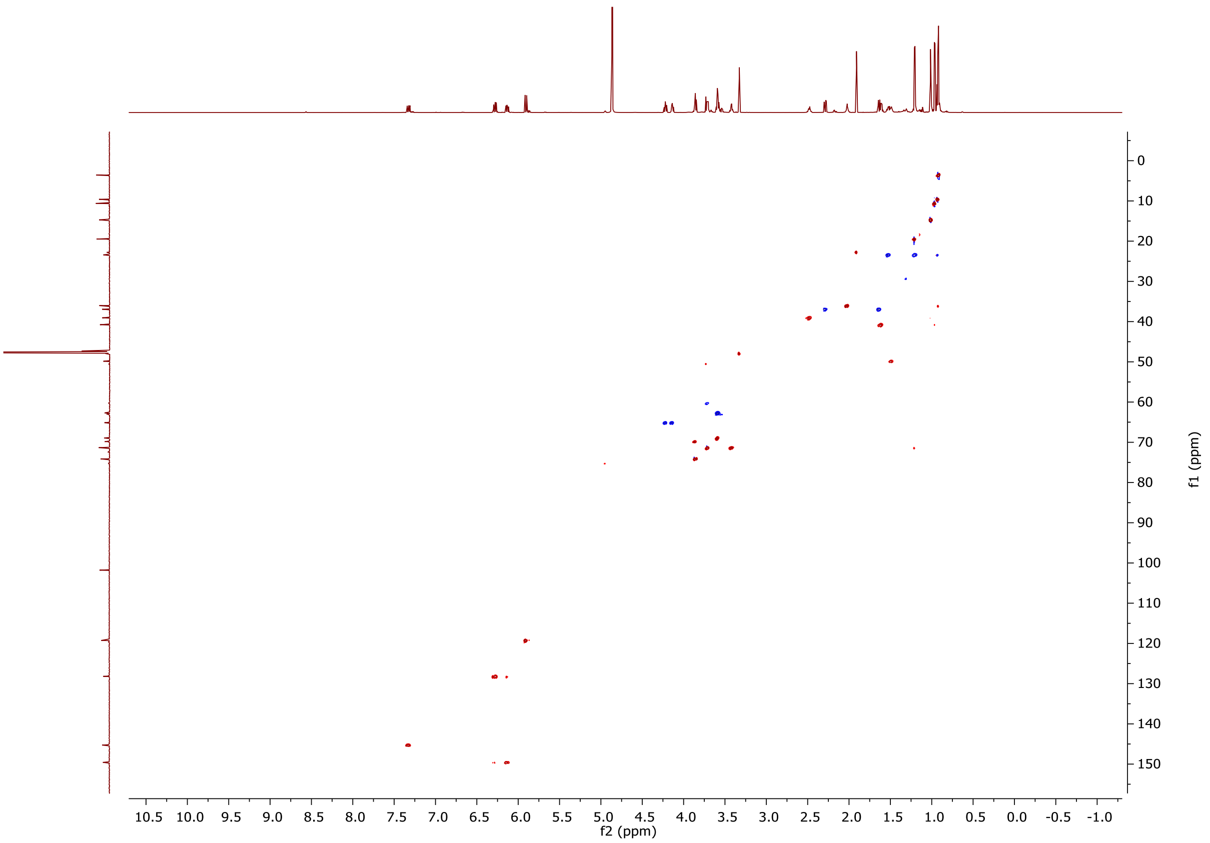

Fig. 8. HSQC spectrum of **1**.

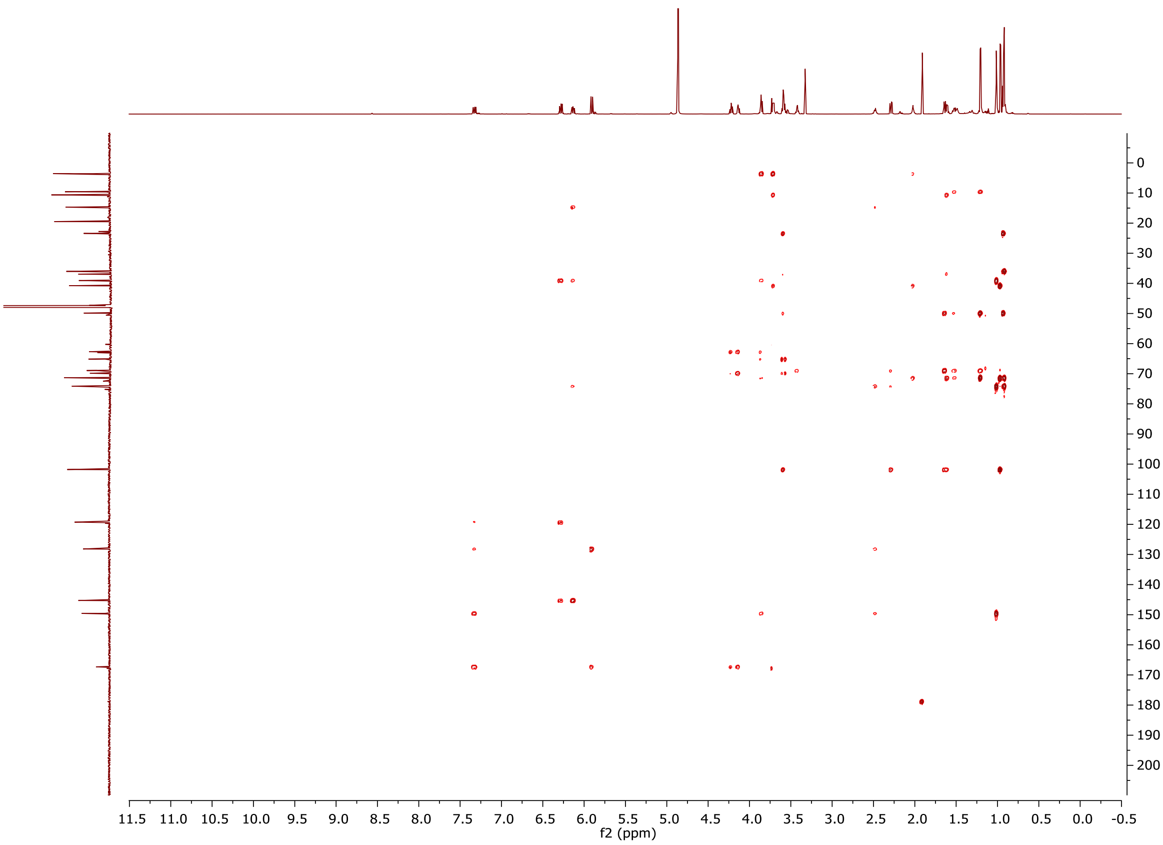

Fig. 9. HMBC spectrum of **1**.

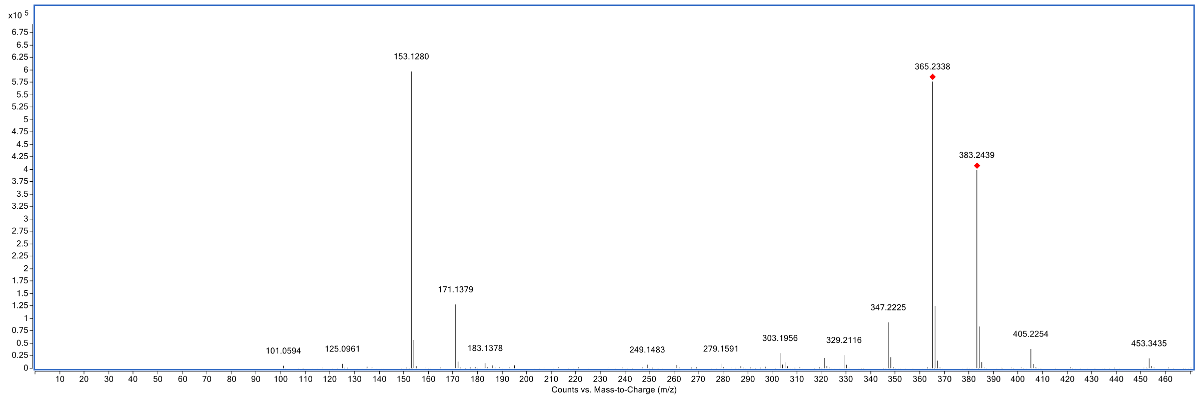

Fig. 10. Mass spectrum of **1**. Its formula of $C_{21}H_{34}O_6$ was deduced by *m/z* 383.2439 $[M+H]^+$ (calculated for 383.2428, *Δ* 2.84 ppm).

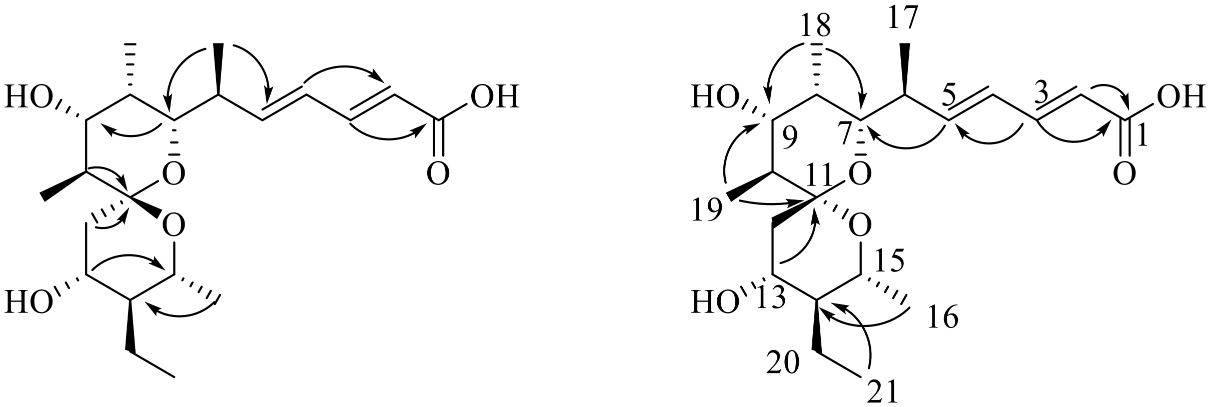

1 **2**

Fig. 11. Selected HMBC correlations for **1** and **2**.

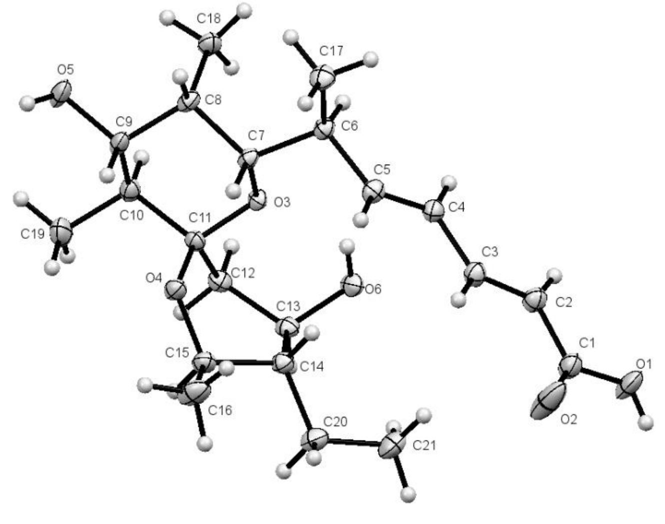

Fig. 12. Crystal structure of **1**. ORTEP diagram showing the atom-numbering scheme and solid-state conformation of **1**.

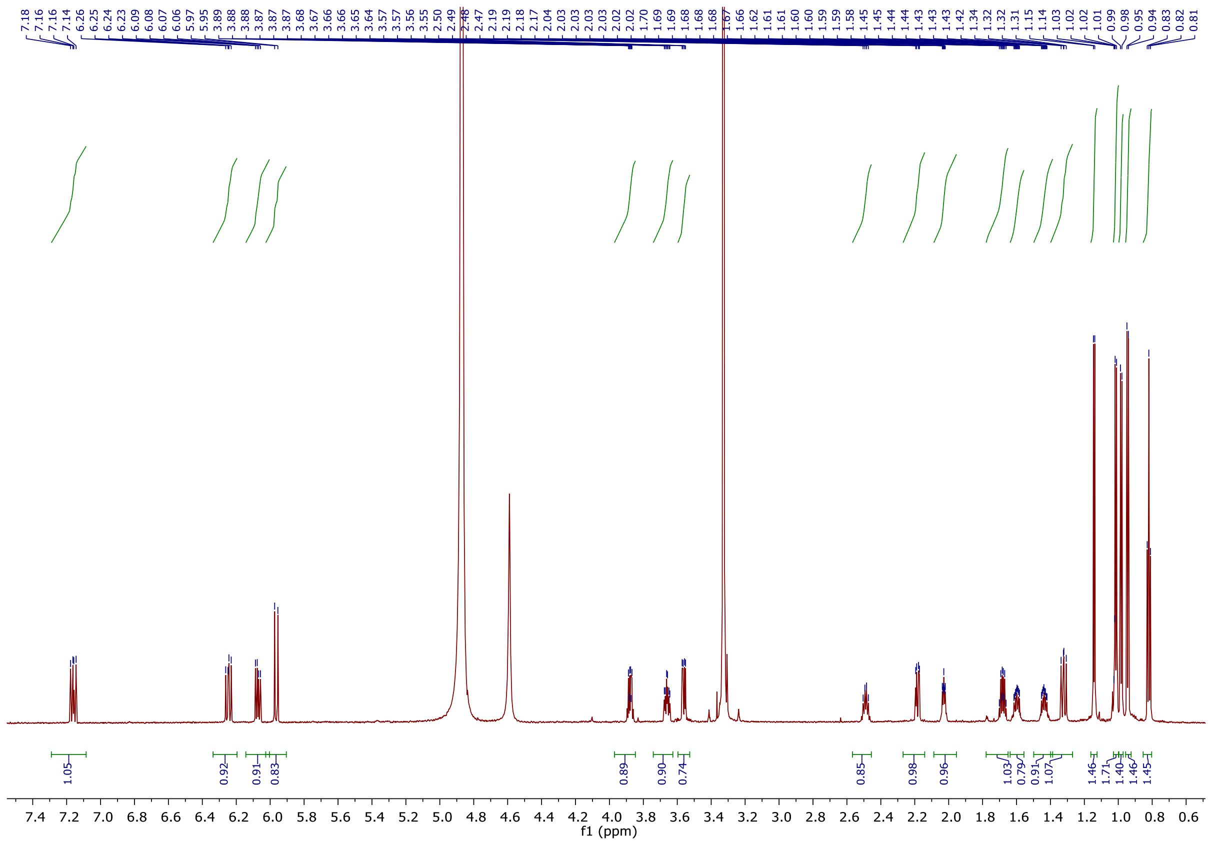

Fig. 13. ^{1}H NMR spectrum of **2**.

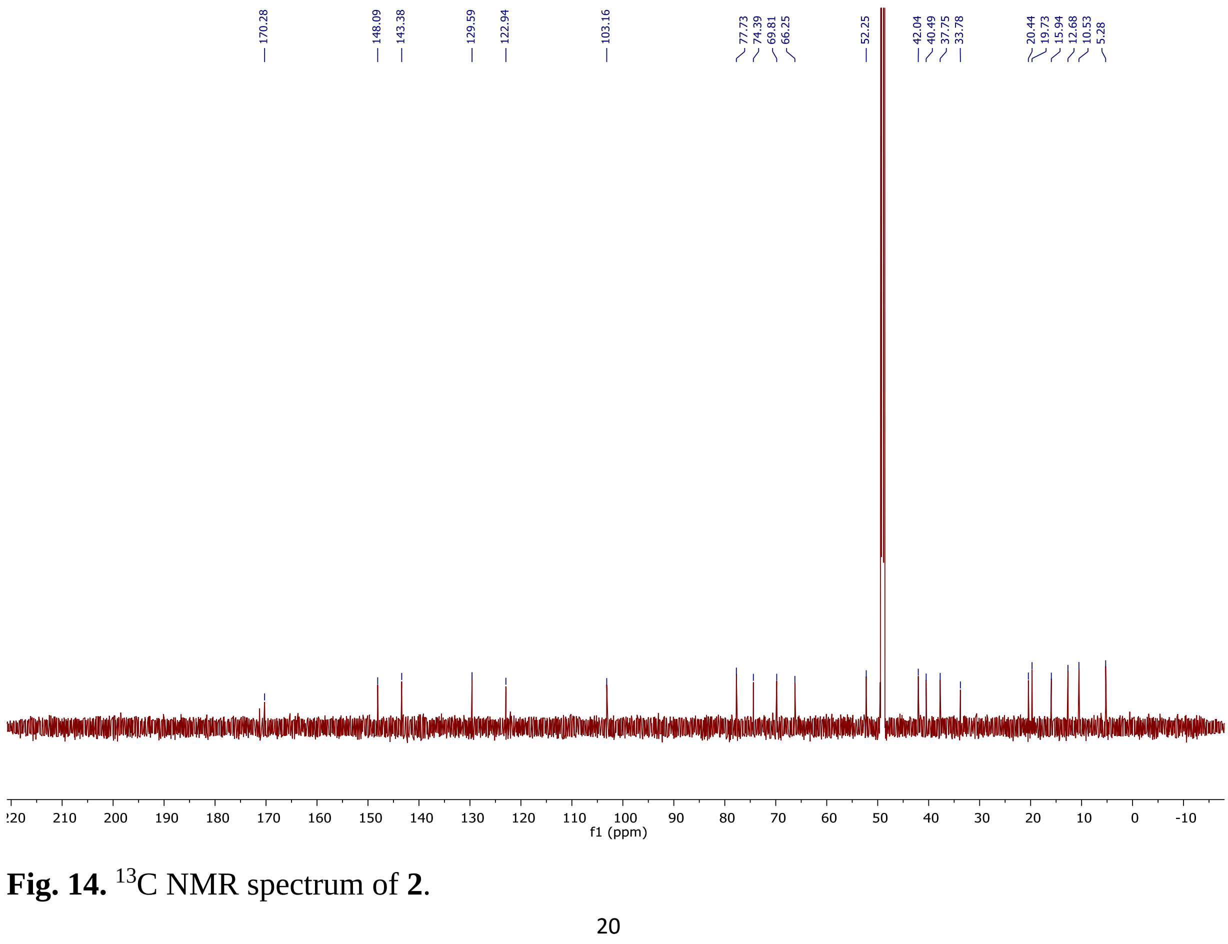

Fig. 14. ^{13}C NMR spectrum of **2**.

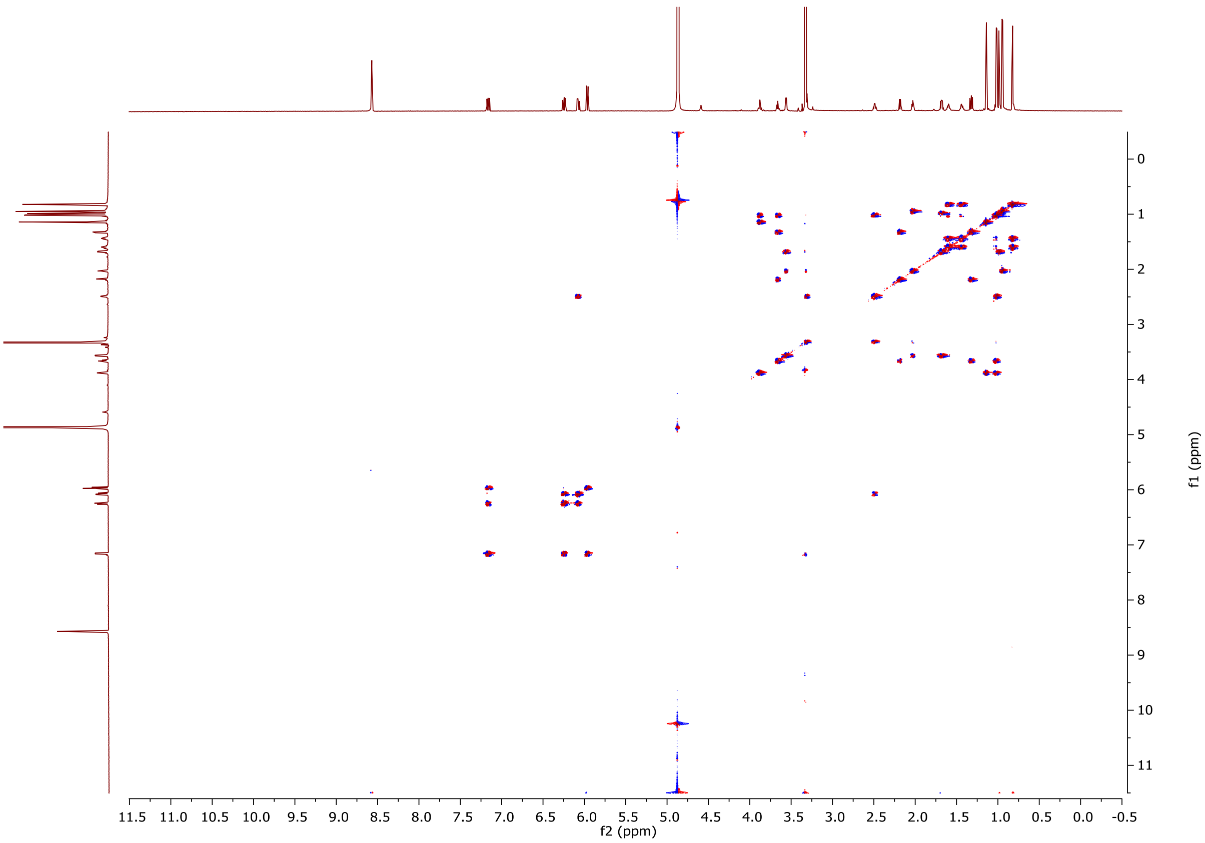

Fig. 15. COSY spectrum of **2**.

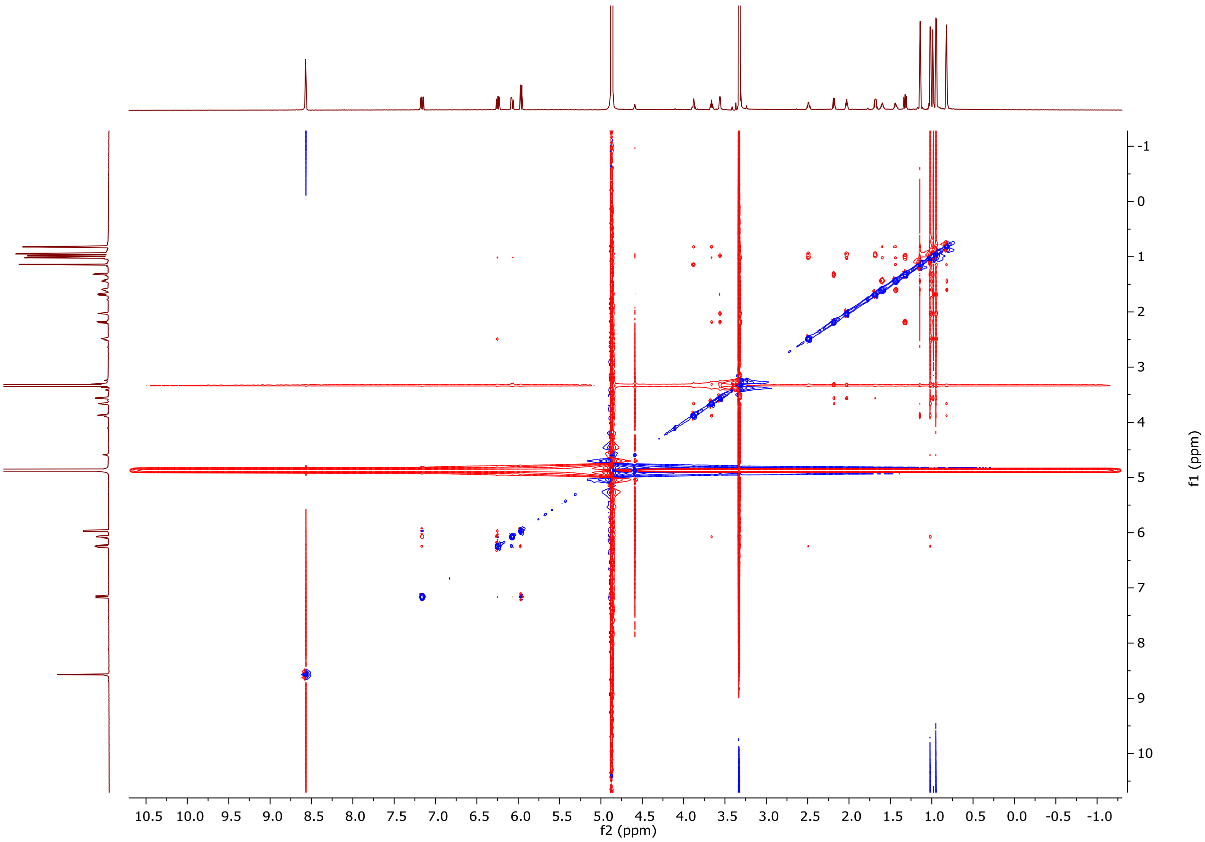

Fig. 16. NOESY spectrum of **2**.

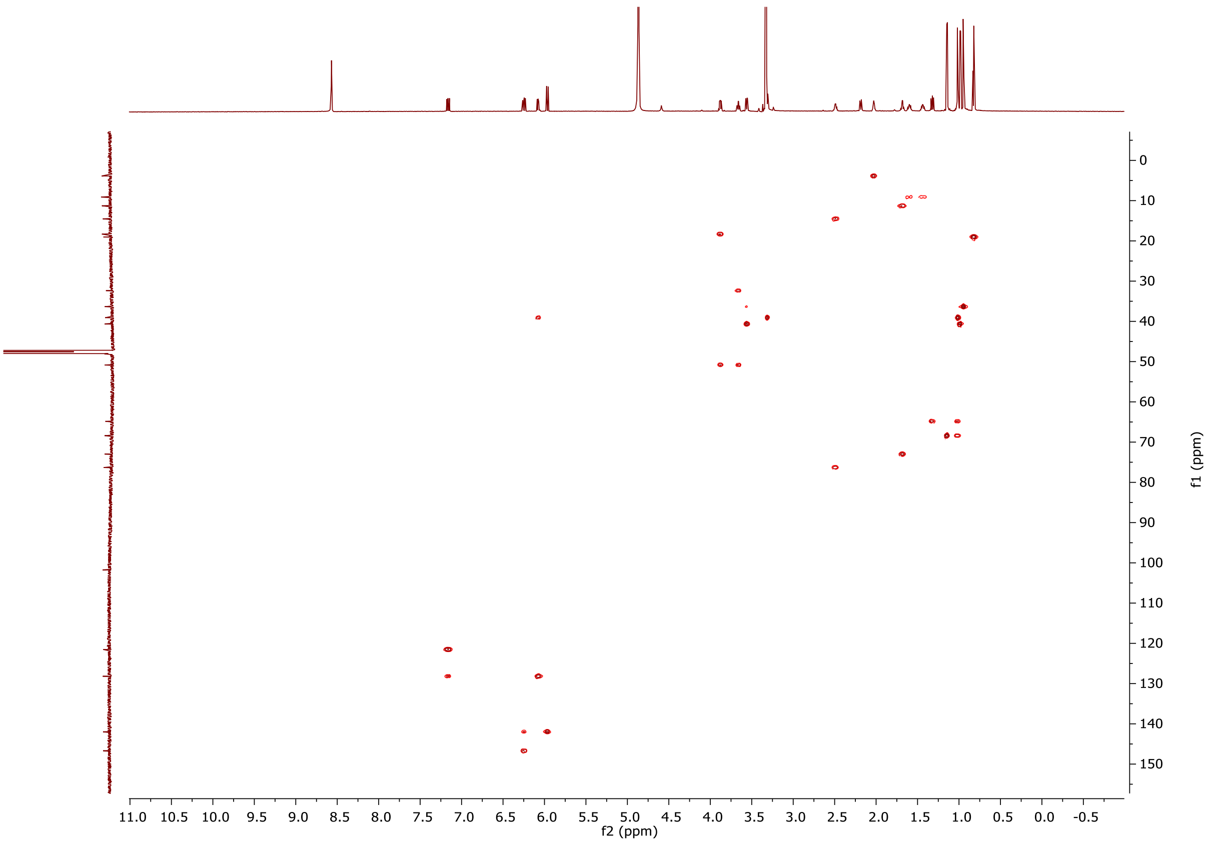

Fig. 17. H2BC spectrum of compound **2**.

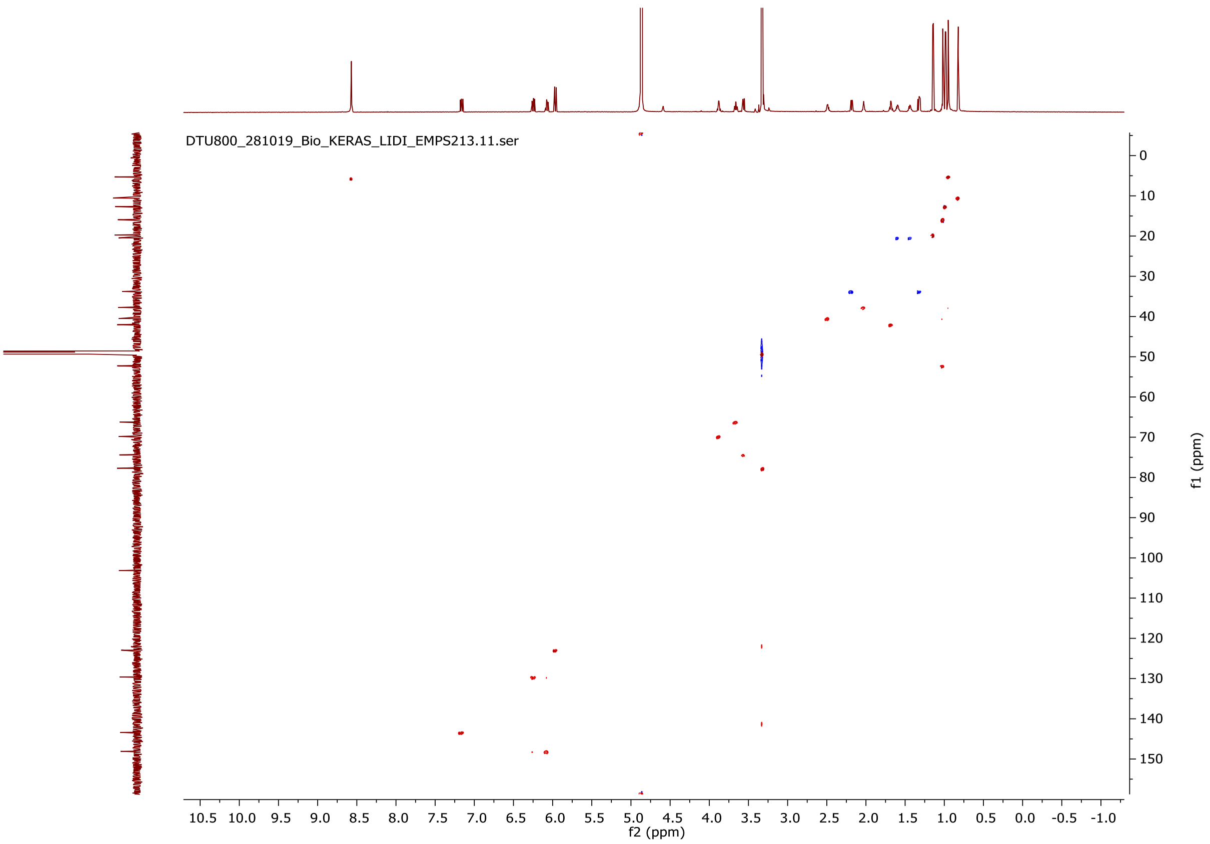

Fig. 18. HSQC spectrum of **2**.

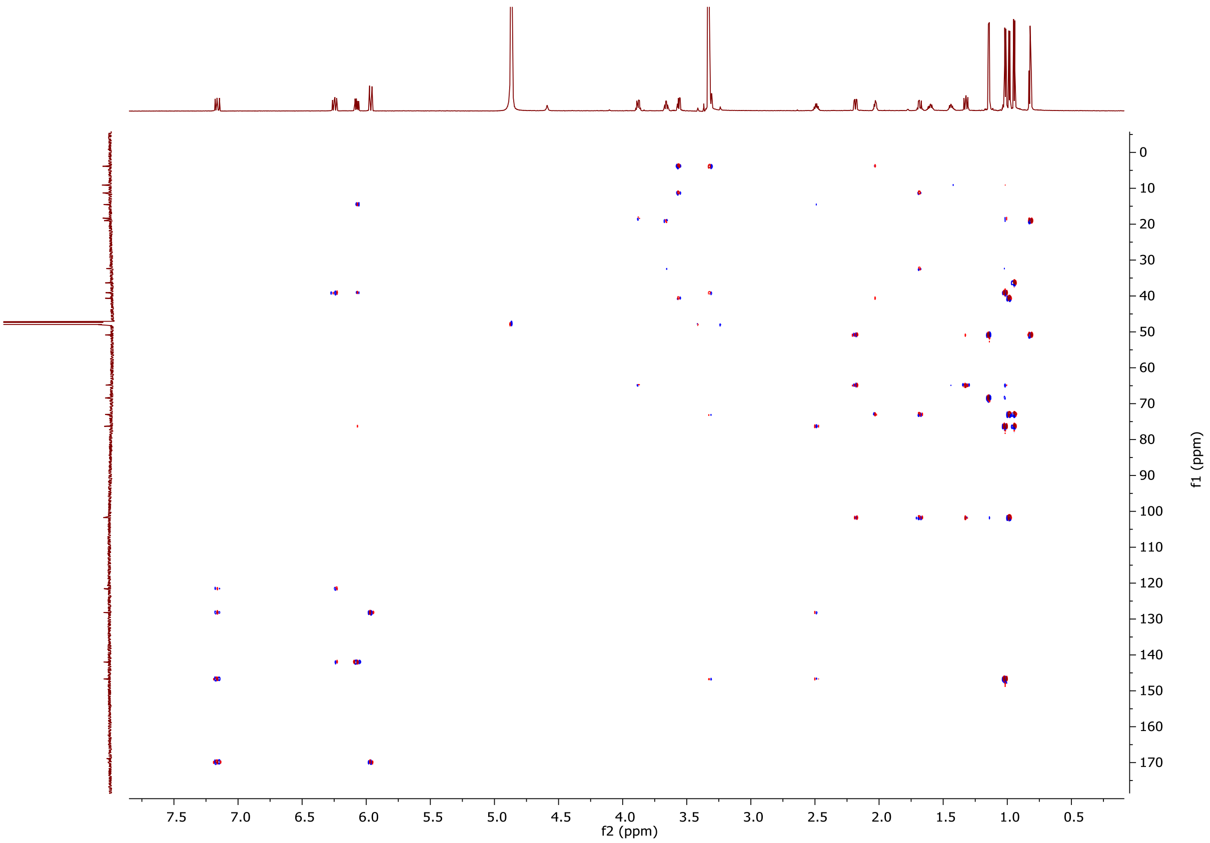

Fig. 19. HMBC spectrum of **2**.

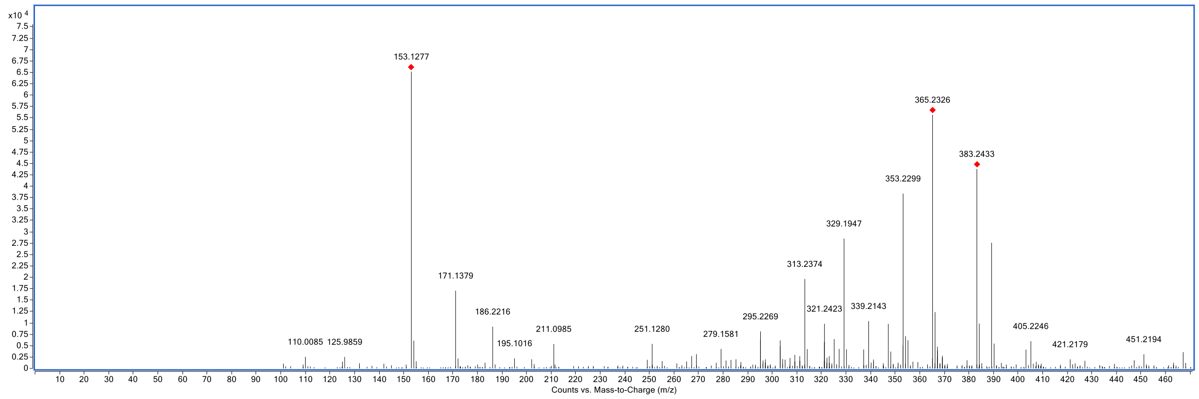

Fig. 20. Mass spectrum of **2**. Its formula of $C_{21}H_{34}O_6$ was deduced by *m/z* 383.2433 $[M+H]^+$ (calculated for 383.2428, *Δ* 1.27 ppm).

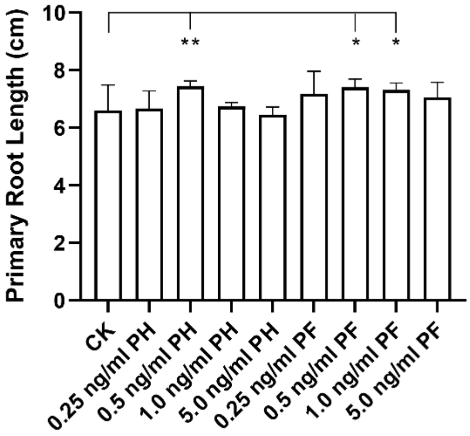

Fig. 21. Primary root length of *Arabidopsis* seedling treated with different concentrations of pteridic acids H and F. Abbreviation: *CK*, blank control treated by sterile Milli-Q water; *PH*, treatment of pteridic acid H; *PF*, treatment of pteridic acid F (mean ± SD, n=16). Statistical significance was assessed by T-test. Asterisks indicate the level of statistical significance: $^{*}p < 0.05$, $^{**}p < 0.01$. Source data are provided as a Source Data file.

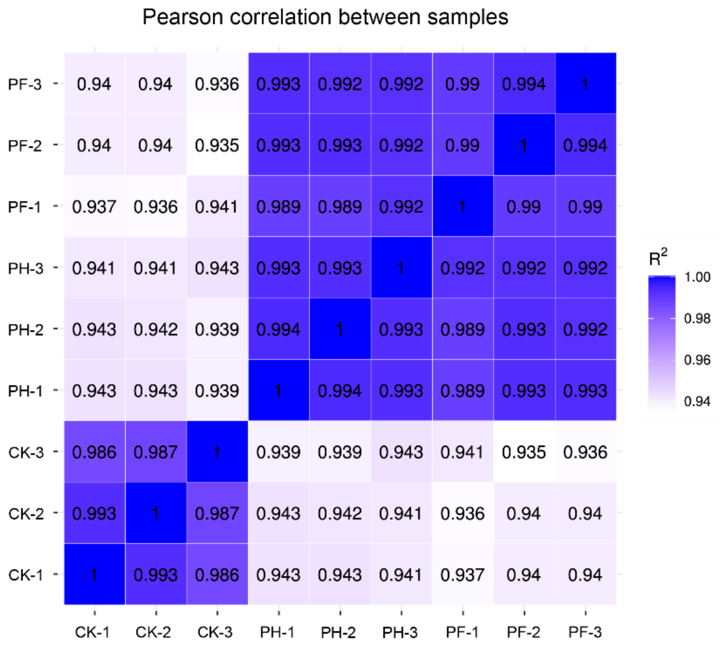

Fig. 22. Inter-sample correlation heat map. R^2: Square of Pearson correlation coefficient (R). Abbreviation: *PH*, treatment of pteridic acid H in *Arabidopsis* seedlings under NaCl-mediated salt stress; *PF*, treatment of pteridic acid F in *Arabidopsis* seedlings under NaCl-mediated salt stress; *CK*, treatment of equal water in *Arabidopsis* seedlings under NaCl-mediated salt stress as control.

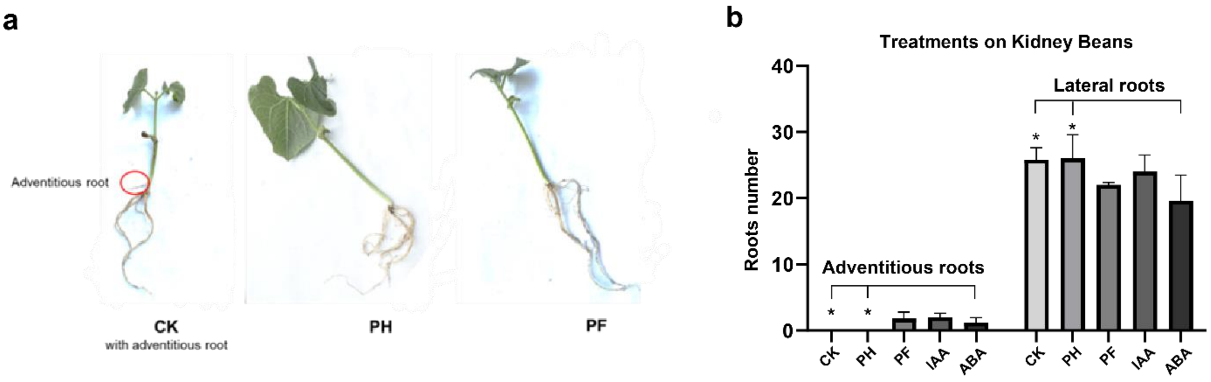

Fig. 23. Kidney beans growth experiment with pure pteridic acids. ***a***, the phenotypes of Kidney beans after treatments with pteridic acids H and F. ***b***, the numbers of adventitious roots and lateral roots of Kidney beans after different treatments (mean ± SD, n=5). Abbreviation: *CK*, control; *PH*, treatment of 1 ng mL^{-1} pteridic acid H; *PF*, treatment of 1 ng mL^{-1} pteridic acid F; *IAA*, treatment of 1 ng mL^{-1} IAA; *ABA*, treatment of 1 ng mL^{-1} ABA. Asterisks indicate the level of statistical significance: $^{*}p < 0.05$. Statistical significance was assessed by T-test. Source data are provided as a Source Data file.

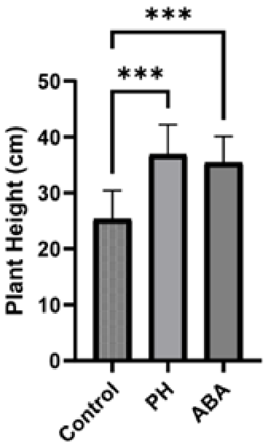

Fig. 24. Pteridic acid H and ABA at 1 ng mL^{-1} help Mung beans against heavy metal stress. The plant height of Mung beans after treatments with pteridic acids H and ABA. Abbreviation: *CK*, Control; *PH*, treatment of pure pteridic acid H; *ABA*, treatment of abscisic acid (mean ± SD, n=9). Statistical significance was assessed by one-way ANOVA with post hoc Dunnett's multiple comparisons test. Asterisks indicate the level of statistical significance: $^{***}p < 0.001$. Source data are provided as a Source Data file.

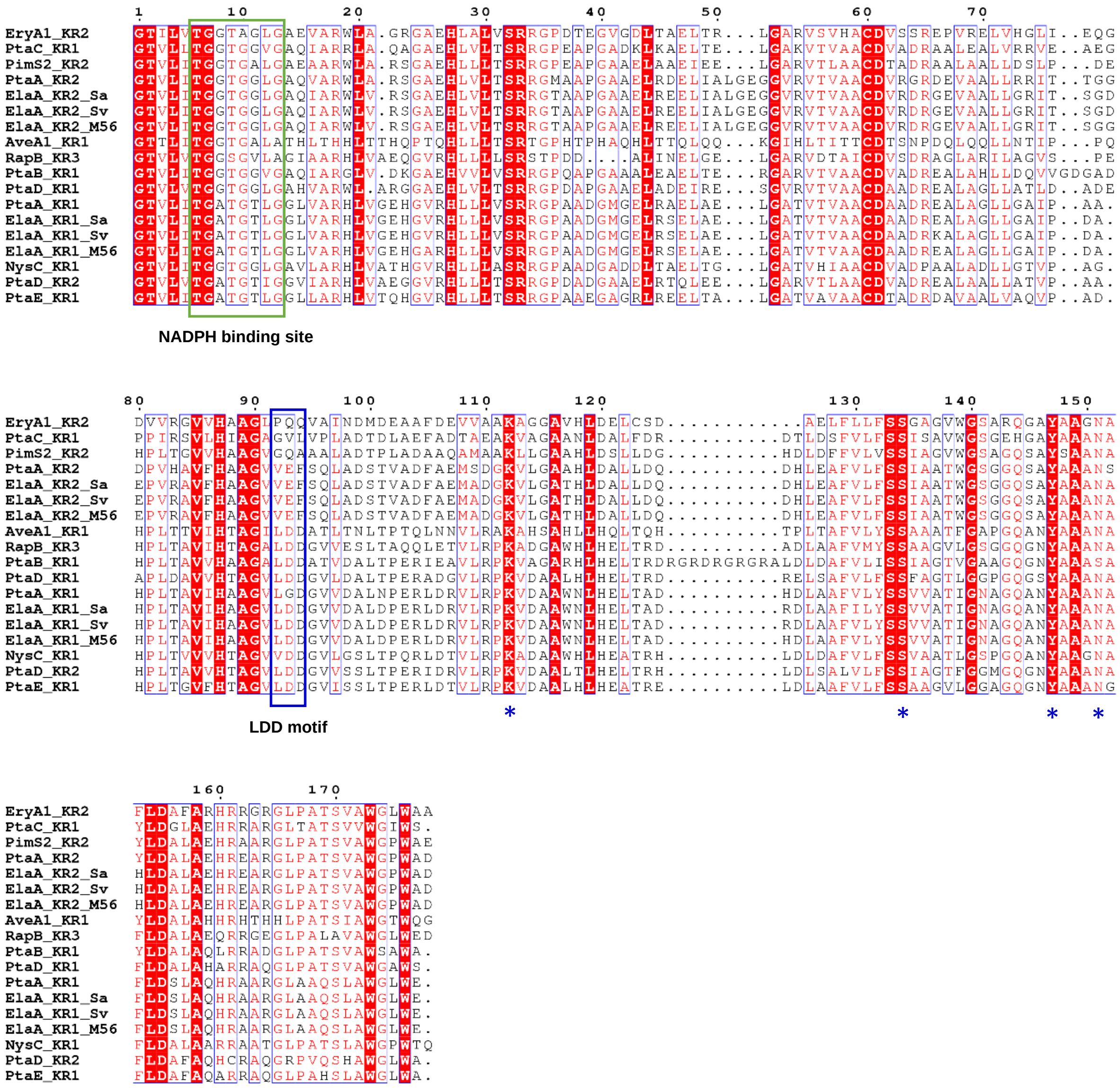

Fig. 25. Multiple sequence alignment of KR domains. The green box indicates NADPH binding site and blue box indicates the LDD motif. Abbreviation: Ery, erythromycin; Pta, pteridic acids; Pim, pimaricin; Ave, avermectin; Rap, rapamycin; Nys, nystatin.

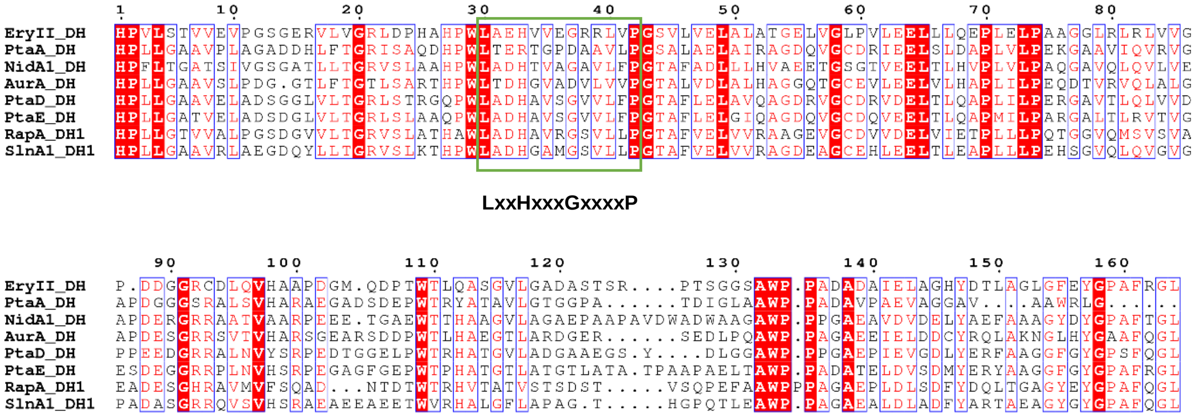

Fig. 26. Multiple sequence alignment of DH domains. The green box indicates the conserved LxxHxxGxxxxP motif. Abbreviation: Ery, erythromycin; Pta, pteridic acids; Nid, niddamycin; Aur, aureothin; Rap, rapamycin; Sln, salinomycin.

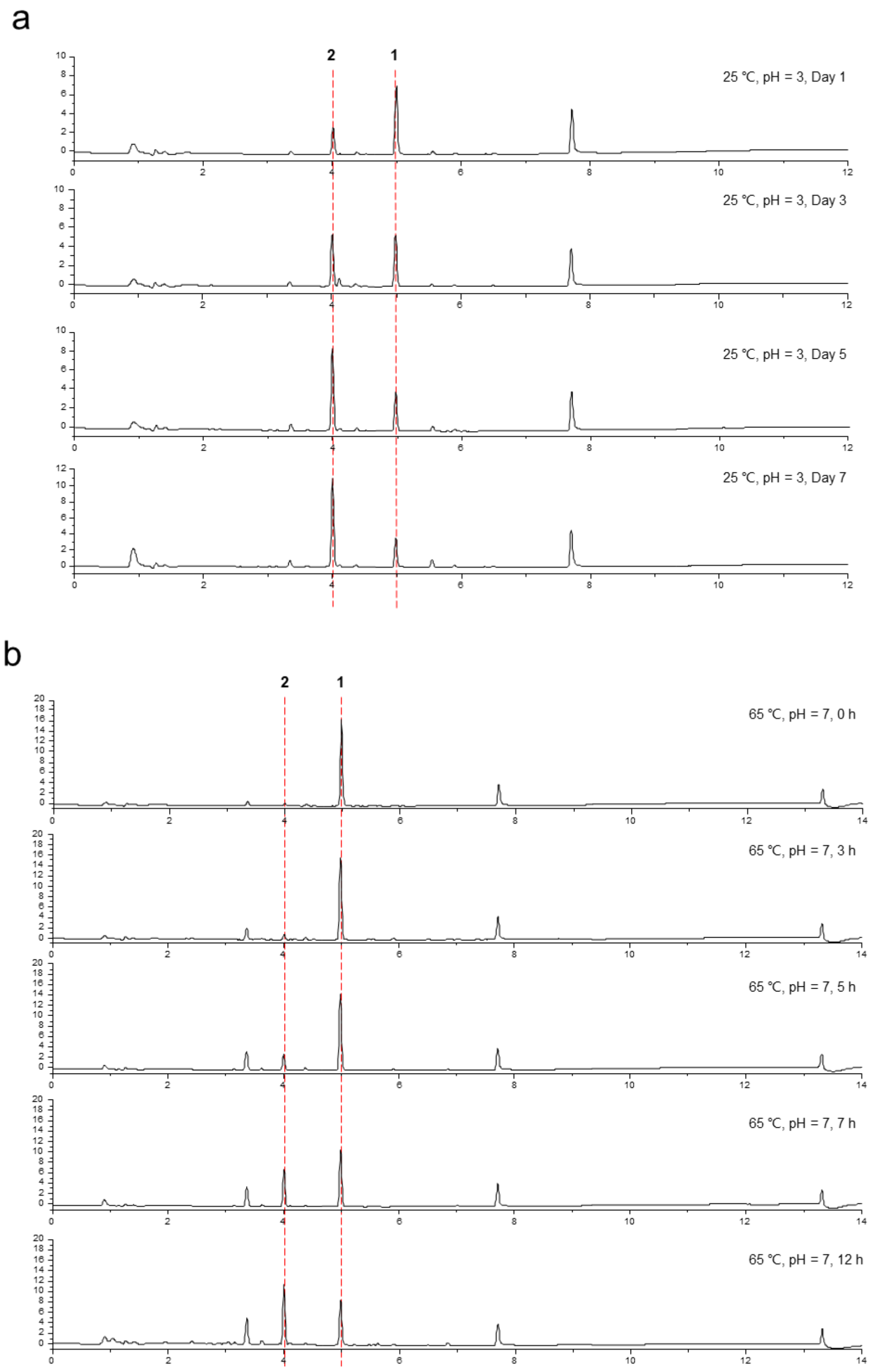

Fig. 27. Stability test of pteridic acids. Abbreviation: **1**, pteridic acid H; **2**, pteridic acid F. **1** was tested unstable in pH 3 buffer solution at 25 ˚C. ***a***, **1** was transformed to **2** fast, after 3 days the contents of them were as equal. The transformation rate is approximately 12.5%, 25%, 37.5%, 50% in 1 d, 3 d, 5 d and 7 d in pH 3 buffer solution at 25 ˚C. ***b***, **1** was unstable in water with 65 ˚C and transformed to **2** fast, after 12 hours, the content of **1** exceeded **2**. The transformation rate is approximately 7 %, 33 % and 47 % in 3 h, 7 h and 12 h, 65 ˚C.

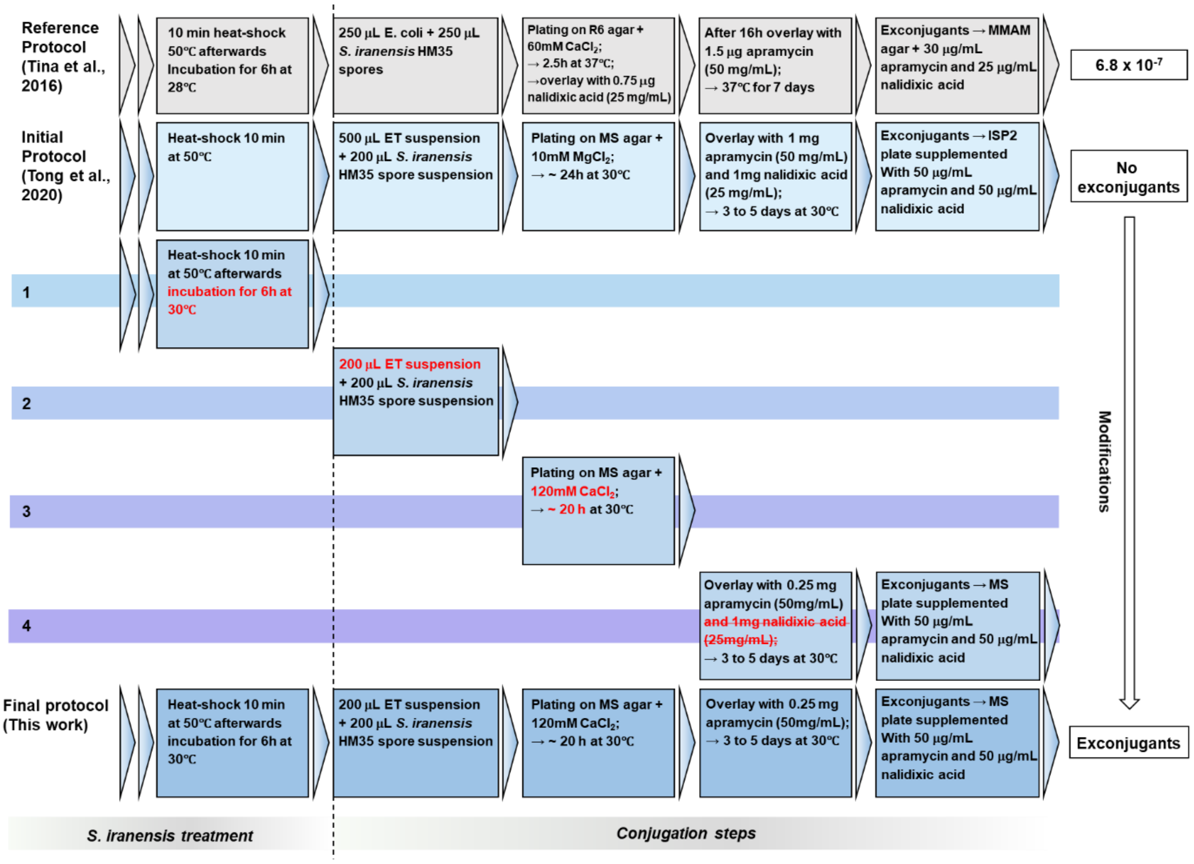

Fig. 28. The schematic of optimized genetic manipulation in *S. iranensis* by using CRISPR-cBEST system.

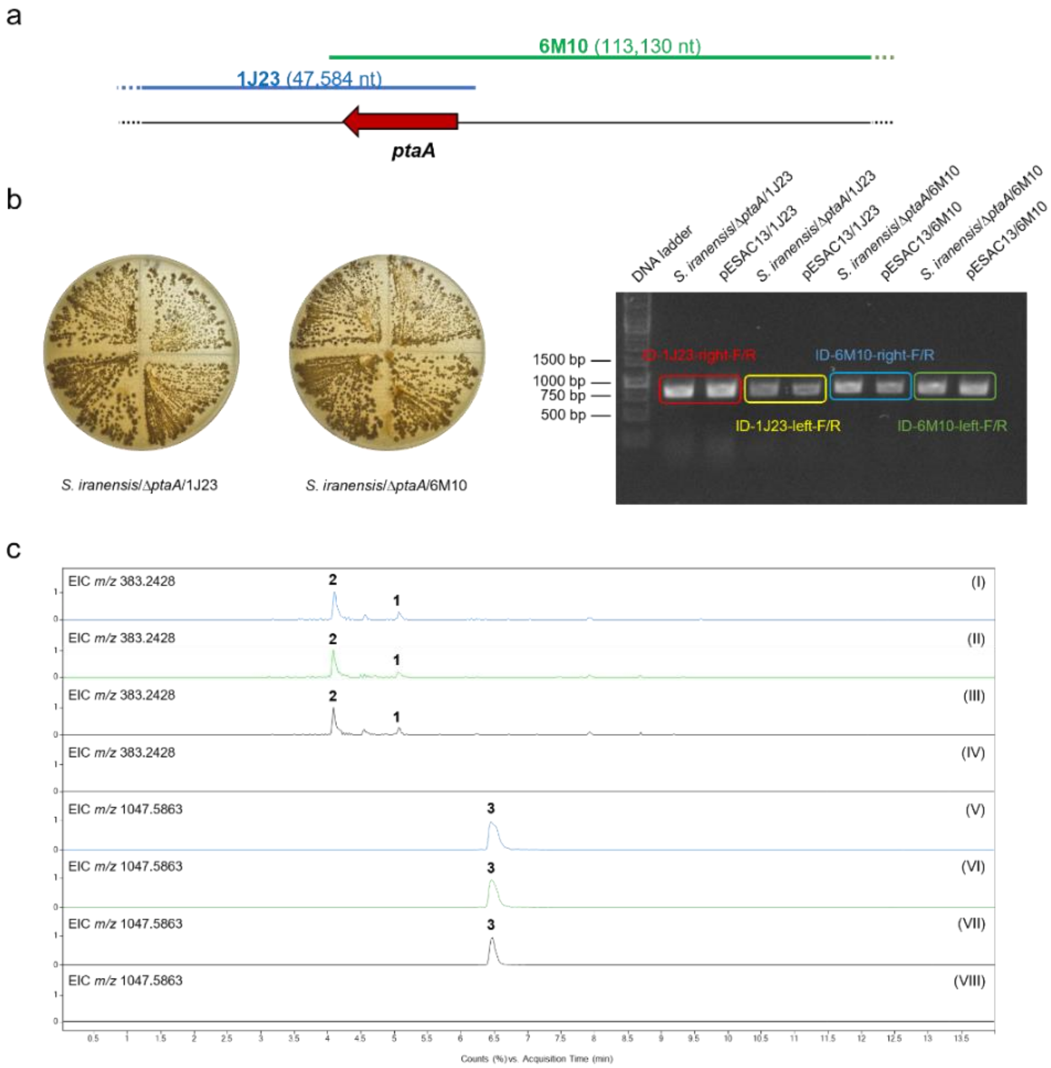

Fig. 29. Complementation experiment of *ptaA*-inactivation mutant of *S. iranensis*. ***a***, location of pESCA13/1J23 and pESCA13/6M10 in genome of *S.iranensis*. ***b***, apramycin-resistance screening and PCR verification of *S. iranensis*/Δ*ptaA*/1J23 and *S. iranensis*/Δ*ptaA*/6M10. ***c***, Extract Ion Chromatography (EIC) in positive mode was performed to detect pteridic acid H (1) and pteridic acid F (2) (*m/z* 383.2428 [M+H]$^+$ $\Delta \pm 5$ ppm) as well as elaiophylin (3) (*m/z* 1047.5863 [M+Na]$^+$ $\Delta \pm 5$ ppm) in the *S. iranensis*/Δ*ptaA*/1J23 (trace I and V), *S. iranensis*/Δ*ptaA*/6M10 (trace II and VI), wild-type *S. iranensis* (trace III and VII), and *S. iranensis*/Δ*ptaA* (trace IV and VIII).

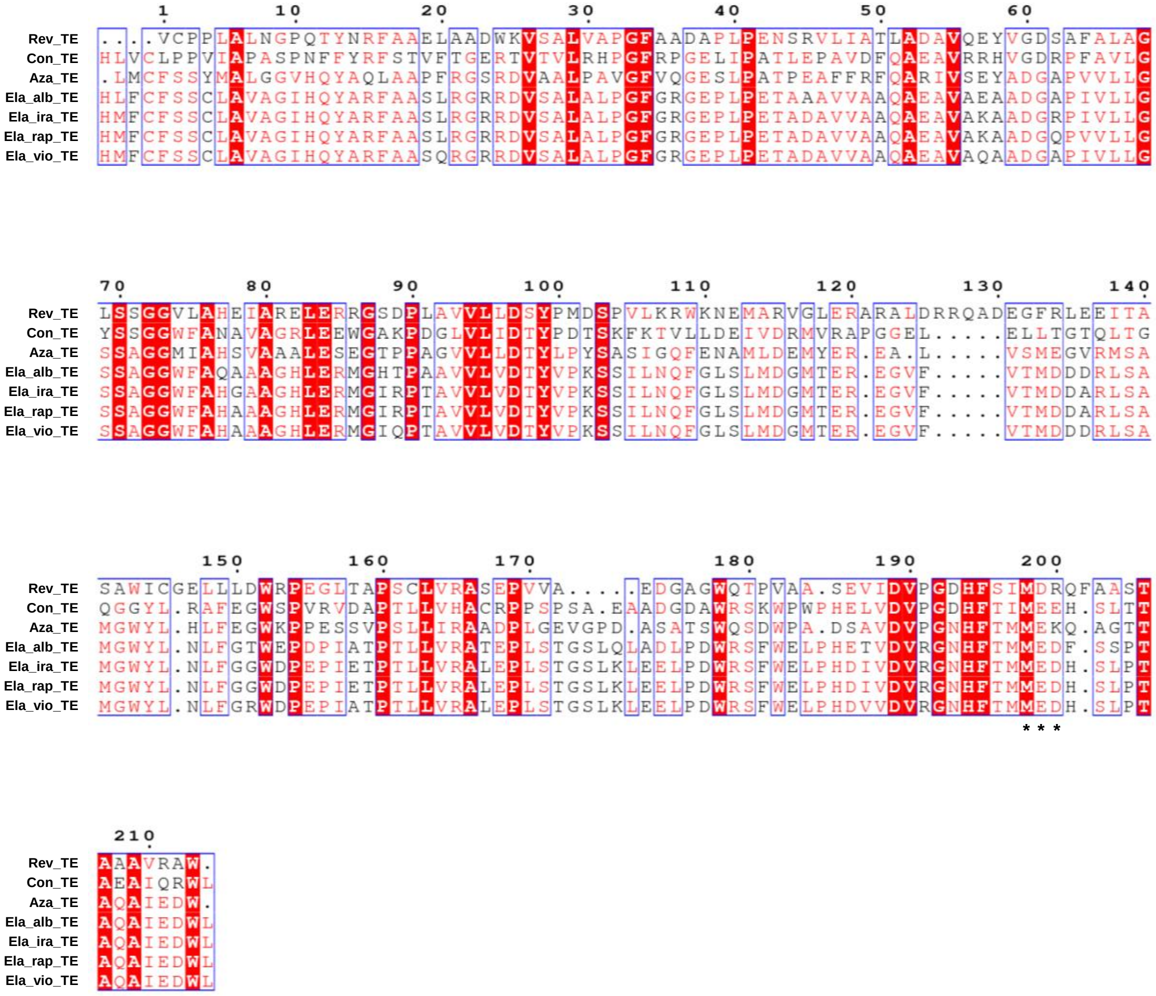

Fig. 30. Multiple sequence alignment of TE domains. The three amino acids marked with an asterisk were mutated in this study. Abbreviation: Rev_TE, the TE domain in reveromycin A biosynthesis from *Streptomyces* sp. SN-593; Con_TE, the TE domain in conglobatin biosynthesis from *Streptomyces conglobatus*; Aza_TE, the TE domain in azalomycin F3a biosynthesis from *Streptomyces* sp. 211726; Ela_alb_TE, the TE domain in elaiophylin biosynthesis from *S. albus* DSM 41398; Ela_ira_TE, the TE domain in elaiophylin biosynthesis from *S. iranensis* HM 35; Ela_rap_TE, the TE domain in elaiophylin biosynthesis from *S. rapamycinicus* NRRL 5491; Ela_vio_TE, the TE domain in elaiophylin biosynthesis from *S. violaceusniger* Tu 4113.

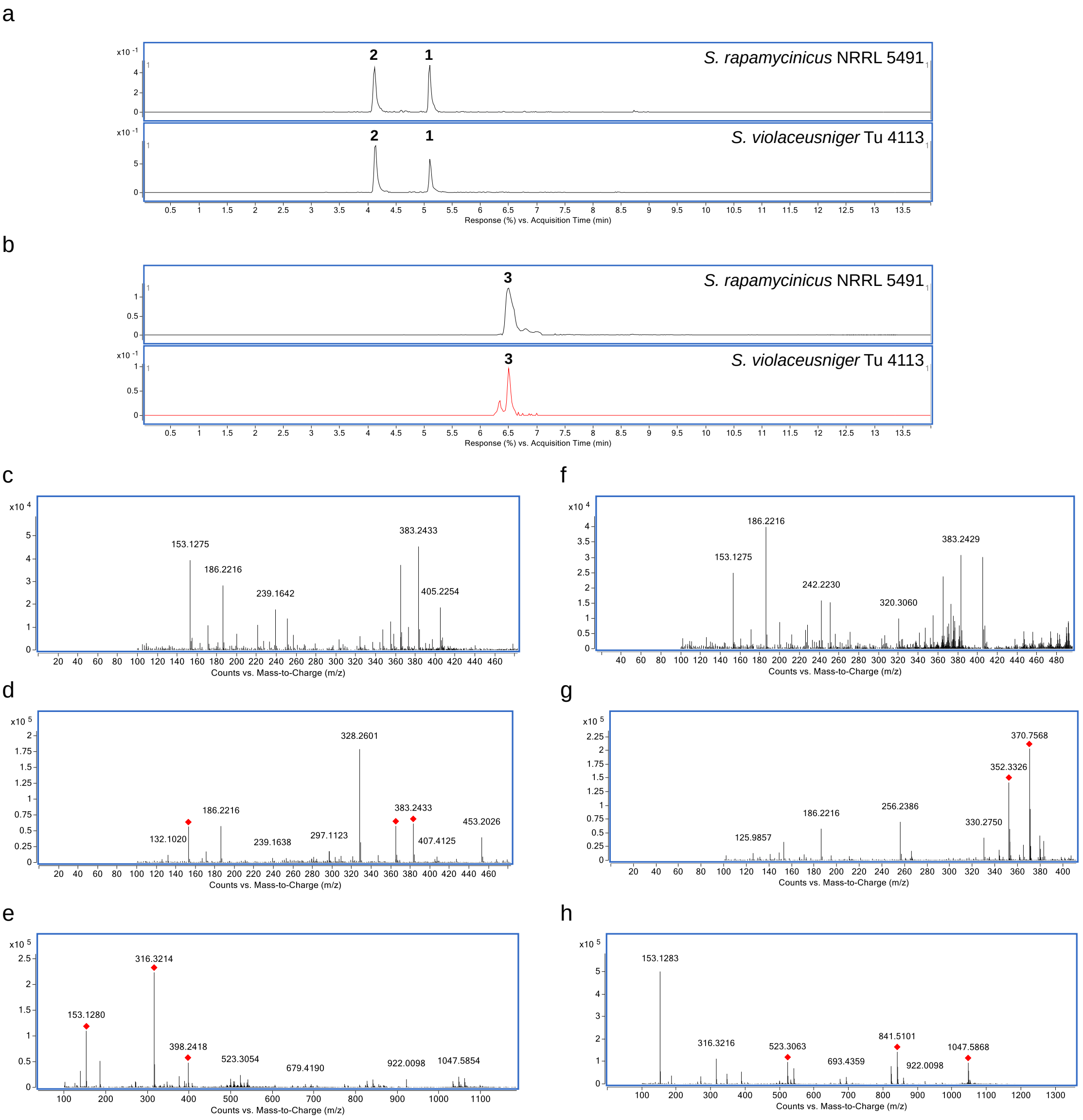

Fig. 31. The HR-LC-MS analysis of metabolites in *S. rapamycinicus* NRRL 5491 and *S. violaceusniger* Tu 4113. ***a***, the Extracted Ion Chromatography (EIC) of *m/z* 383.2428 $[M+H]^+$ in wild-type *S. rapamycinicus* NRRL 5491 and *S. violaceusniger* Tu 4113. The single *m/z* expansion for the chromatogram is ± 5 ppm. ***b***, the EIC of *m/z* 1047.5863 $[M+Na]^+$ in wild-type *S. rapamycinicus* NRRL 5491 and *S. violaceusniger* Tu 4113. The single *m/z* expansion for the chromatogram is ± 5 ppm. ***c***, the HR-LC-MS spectrum of **1** in wild-type *S. violaceusniger* Tu 4113. ***d***, the HR-LC-MS spectrum of **1** in wild-type *S. rapamycinicus* NRRL 5491. ***e***, the HR-LC-MS spectrum of **2** in wild-type *S. violaceusniger* Tu 4113. ***f***, the HR-LC-MS spectrum of **2** in wild-type *S. rapamycinicus* NRRL 5491. ***g***, the HR-LC-MS spectrum of **3** in wild-type *S. violaceusniger* Tu 4113. ***h***, the HR-LC-MS spectrum of **3** in wild-type *S. rapamycinicus* NRRL 5491.

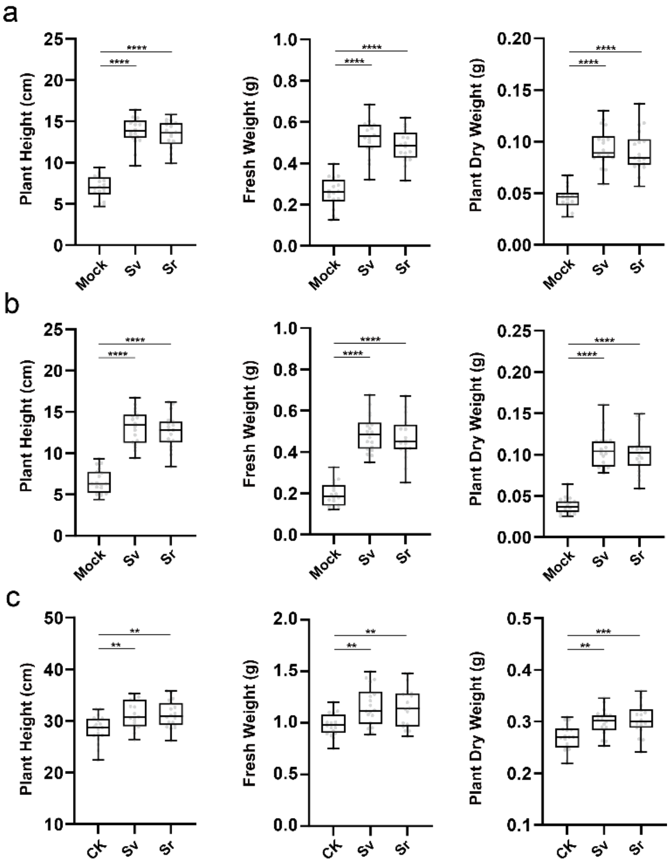

Fig. 32. Abiotic stresses alleviation led by *S. violaceusniger* Tu 4113 and *S. rapamycinicu*s NRRL 5491. ***a***, different growth of barley seedlings in osmotic stress mediated by 20% (w/v) PEG-6000 (data is mean ± SD, n=16). Abbreviation: *Mock*, control; *Sv*, treatment of *S. violaceusniger* Tu 4113 culture broth; *Sr*, treatment of *S. rapamycinicus* NRRL 5491 culture broth; *b*, different growth of barley seedlings in salinity stress mediated by 100 mM NaCl (mean ± SD, n=16). Abbreviation: *Mock*, control; *Sv*, treatment of *S. violaceusniger* Tu 4113 culture broth; *Sr*, treatment of *S. rapamycinicus* NRRL 5491 culture broth; *c*, different growth of barley seedlings in drought stress (mean ± SD, n=16). Abbreviation: *CK*, 7 days treatment of water after 7 days water + 7 days drought; *Sv*, 7 days treatment of *S. violaceusniger* Tu 4113 culture broth after 7 days water + 7 days drought; *Sr*, 7 days treatment of *S. rapamycinicus* NRRL 5491 culture broth after 7 days water + 7 days drought. Statistical significance was assessed by one-way ANOVA with post hoc Dunnett's multiple comparisons test. Asterisks indicate the level of statistical significance: *$p < 0.05$, **$p < 0.01$, ***$p < 0.001$ and ****$p < 0.0001$. All box plots with centre lines showing the medians, boxes indicating the interquartile range, and whiskers indicating a range of minimum to maximum data beyond the box. Source data are provided as a Source Data file.

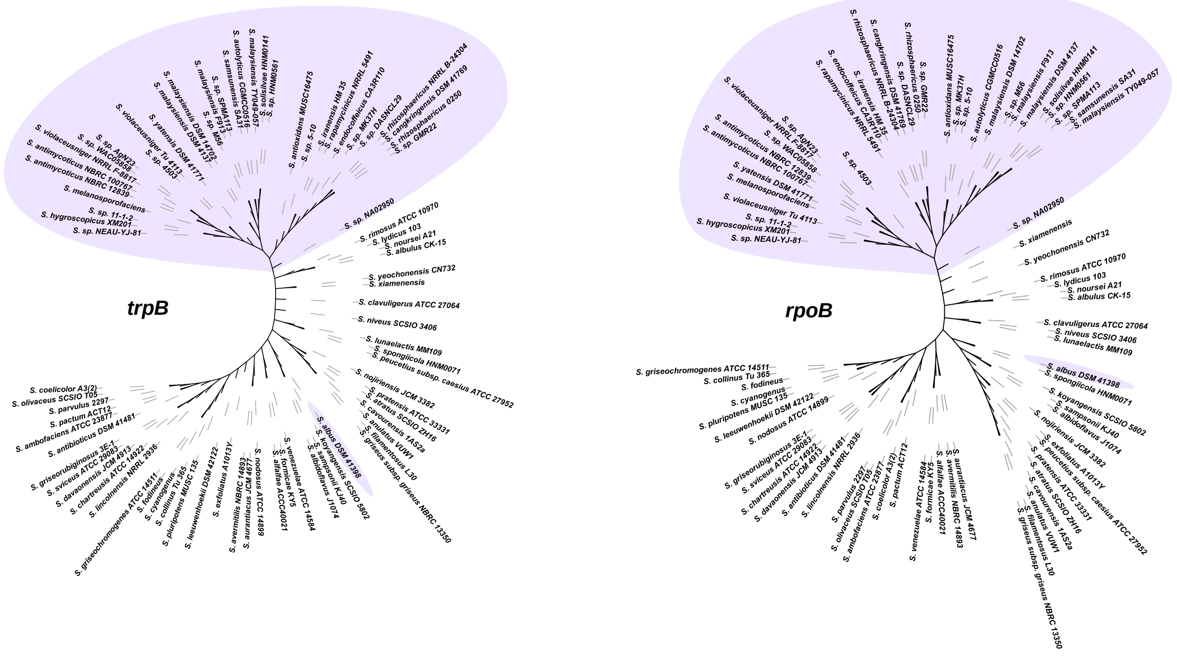

Fig. 33. The phylogenetic analysis of potential pteridic acids *Streptomyces* producers. The high-resolution *Streptomyces* sp. housekeeping genes: *trpB* (tryptophan synthase subunit beta) and *rpoB* (RNA polymerase subunit beta) were used in this analysis.

a

		1	2	3	4	5	6	7	8	9	10	11	12	13	14	15
S. rapamycinus NRRL 5491	1		95.78	83.53	91.79	91.64	91.73	91.65	91.60	91.75	91.72	91.61	91.59	86.13	91.59	91.50
S. iranensis HM 35	2	74.82		83.59	91.80	91.72	91.77	91.68	91.62	91.79	91.63	91.62	91.64	86.10	91.73	91.59
S. albus DSM 41398	3	10.75	10.61		83.56	83.60	83.52	83.57	83.60	83.54	83.54	83.61	83.63	83.70	83.47	83.56
S. antimycoticus NBRC 100767	4	70.38	68.49	11.36		91.65	95.60	91.63	91.58	95.58	97.58	91.58	91.58	86.14	95.59	96.12
S. autolyticus CGMCC0516	5	66.51	64.83	11.31	68.75		91.64	98.77	98.91	91.71	91.52	98.92	98.80	86.07	91.52	91.43
S. hygroscopicus XM201	6	66.63	65.41	11.15	73.49	69.52		91.65	91.61	98.90	95.18	91.61	91.59	86.15	96.12	95.16
S. malaysiensis DSM 4137	7	66.86	64.77	11.14	68.99	83.07	68.79		98.80	91.70	91.48	98.81	98.87	86.05	91.52	91.41
S. solisilvae HNM0141	8	65.56	62.94	10.79	67.61	87.21	67.84	89.60		91.63	91.44	99.99	98.80	86.06	91.46	91.38
S. sp. 11-1-2	9	67.91	66.17	11.21	74.30	70.01	87.13	70.25	69.05		95.17	91.64	91.63	86.14	96.11	95.18
S. sp. AgN23	10	68.18	64.40	11.33	78.86	67.16	70.61	67.94	66.24	72.65		91.44	91.47	86.09	95.22	95.78
S. sp. HNM0561	11	65.39	62.80	10.73	67.47	87.01	67.67	89.40	99.30	68.88	66.11		98.80	86.07	91.46	91.39
S. sp. M56	12	65.15	63.01	10.73	67.12	86.71	67.90	88.11	91.88	68.63	65.97	91.67		86.08	91.47	91.37
S. sp. NA02950	13	41.20	41.77	11.53	43.96	43.33	42.16	42.40	41.15	42.67	43.29	41.09	41.12		86.11	86.19
S. violaceusniger Tu 4113	14	67.65	67.66	11.52	76.23	68.53	73.87	68.27	66.21	75.44	72.44	66.08	66.28	43.88		95.25
S. yatensis DSM 41771	15	70.13	69.72	11.89	80.47	70.49	76.37	71.00	69.76	76.91	74.15	69.62	69.57	45.31	80.17	

Upper: average nucleotide identity **Lower:** alignment percentage

b

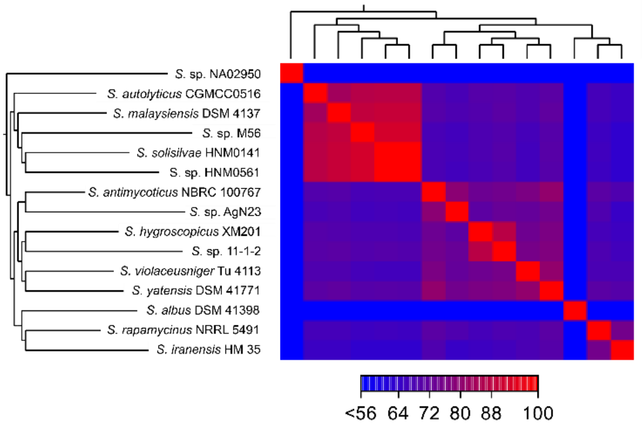

Fig. 34. Genome similarity analysis based on the alignment of 15 sequenced *pta*-containing *Streptomyces* genomes. ***a***, AP (alignment percentage) and ANI (average nucleotide identity) values between genomes. The AP value represents the average percentage of aligned genomic regions between two genomes, whereas the ANI value stands for the percentage of exactly matching nucleotides for these aligned regions. ***b***, Heat map showing genetic similarity between genomes based on AP values.

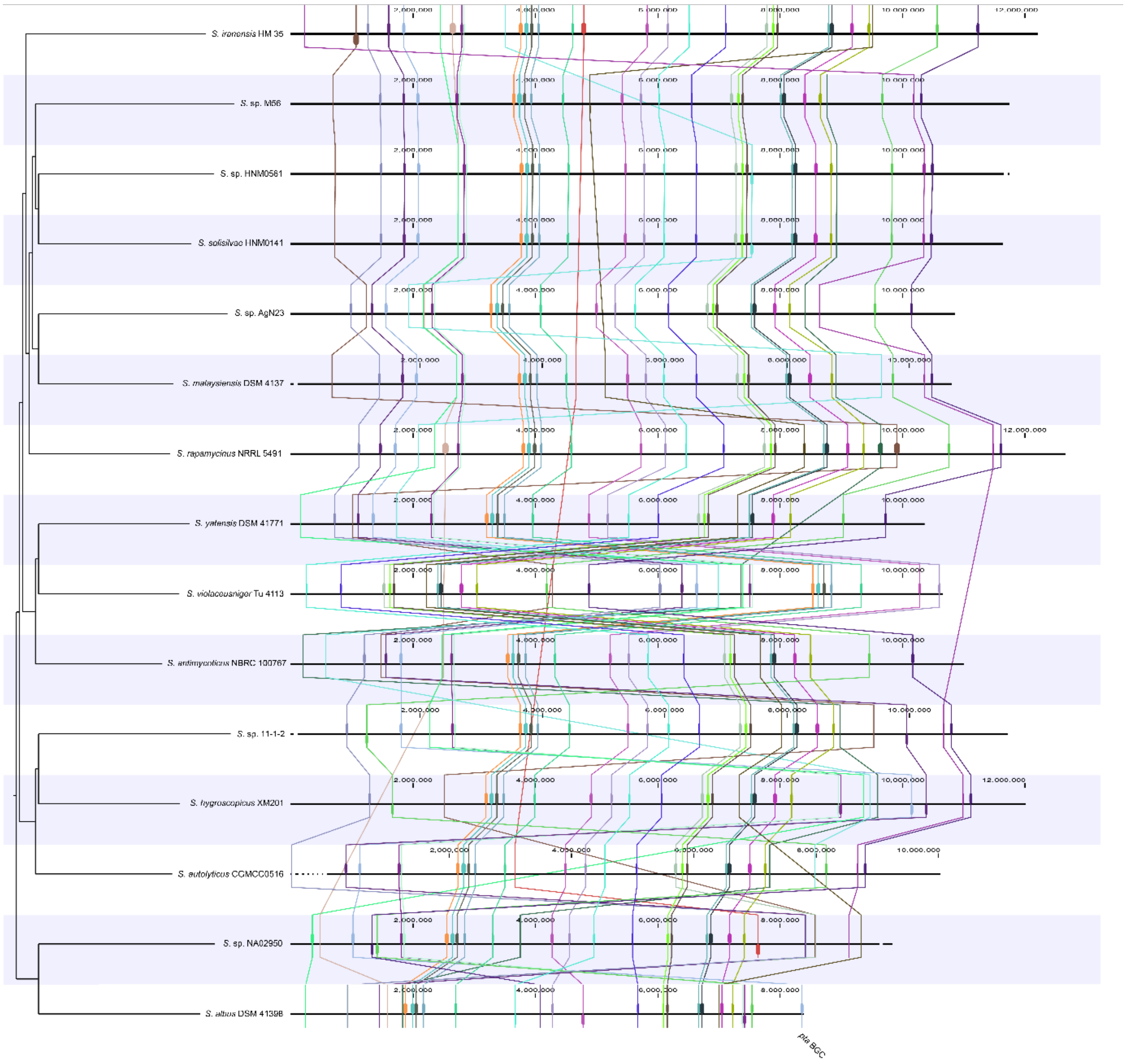

Fig. 35. The genome synteny analysis of selected 15 *pta*-containing *Streptomyces* strains. The colour squares each represent local alignment synteny blocks that are linked together among different genomes by lines with the same colours. The *pta* BGC is at the end of *S. albus* DSM 41398 chromosome.

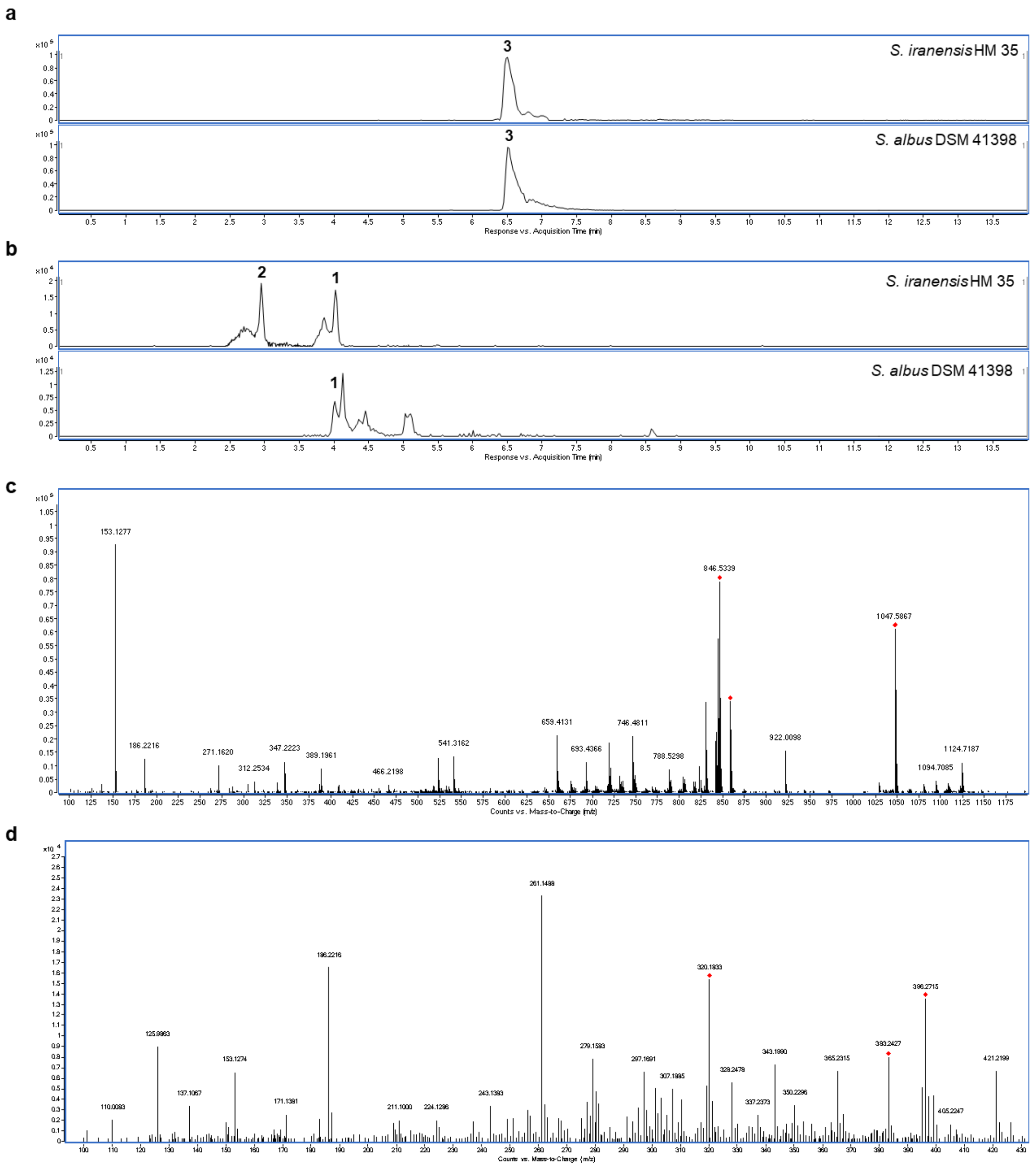

Fig. 36. The HR-LC-MS analysis of metabolites in *S. albus* DSM 41398. ***a***, the EIC of *m/z* 383.2428 $[M+H]^+$ in wild-type *S. iranensis* HM 35 and wild-type *S. albus* DSM 41398. The single *m/z* expansion for the chromatogram is ± 5 ppm. ***b***, the EIC of *m/z* 1047.5863 $[M+Na]^+$ in wild-type *S. iranensis* HM 35 and wild-type *S. albus* DSM 41398. The single *m/z* expansion for the chromatogram is ± 5 ppm. ***c***, the HR-LC-MS spectrum of **3** in wild-type *S. albus* DSM 41398. ***d***, the LC -MS spectrum of **1** in wild-type *S. albus* DSM 41398.

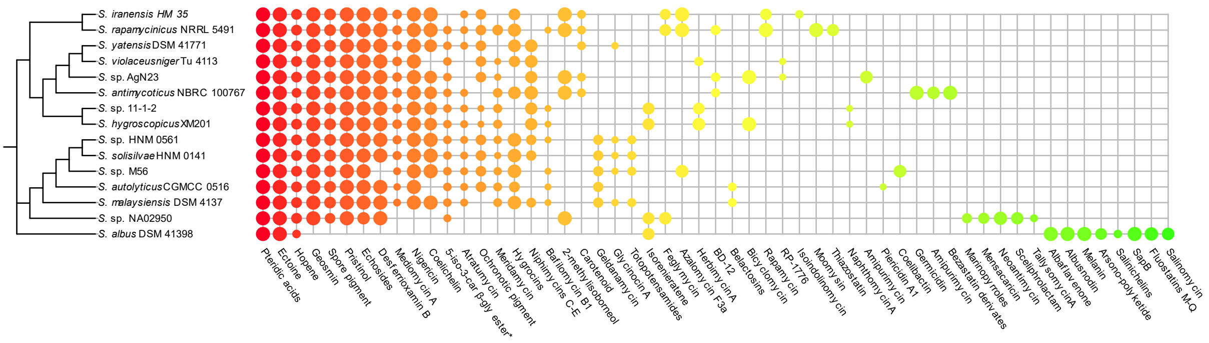

Fig. 37. The secondary metabolites biosynthetic gene clusters profiles of 15 *pta*-containing *Streptomyces* strains with complete genome information. BGCs with a similarity greater than 45% are displayed, and the size of the nodes represents the level of similarity *, 5-isoprenylindole-3-carboxylate β-glycosyl ester.

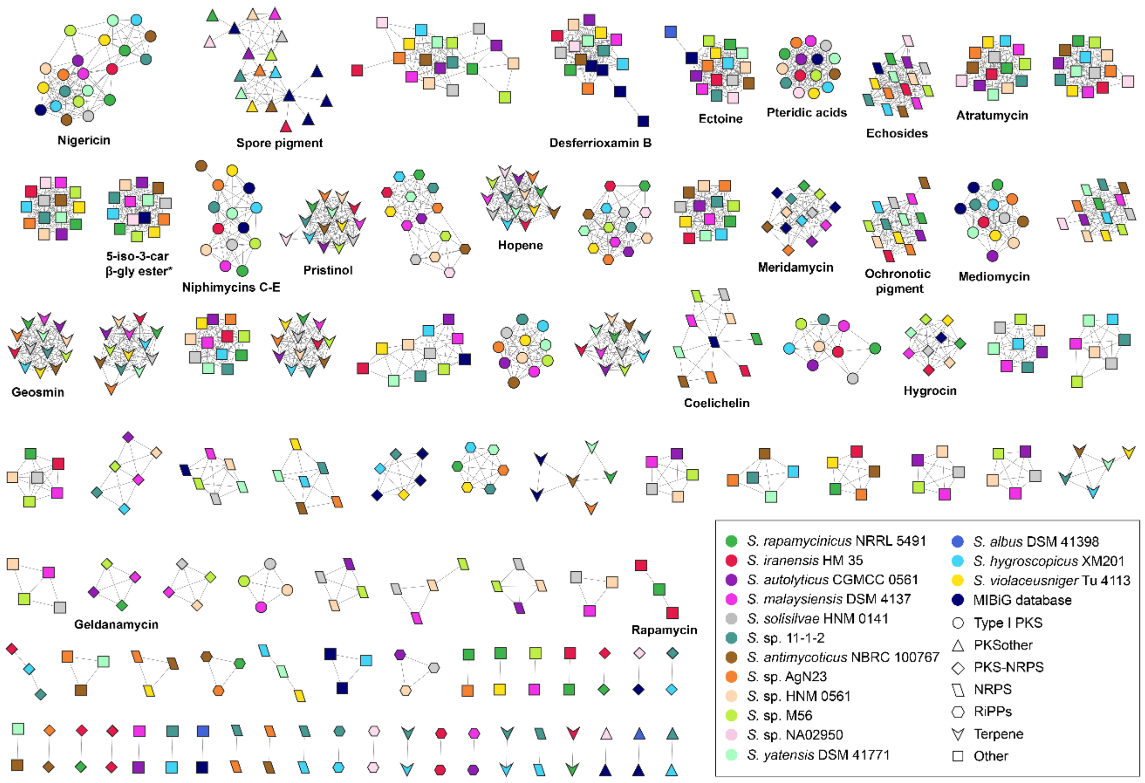

Fig. 38. The secondary metabolites BGCs similarity network of 15 *pta*-containing *Streptomyces* strains with complete genome information. Different strains and types of secondary metabolites are represented by different colours and shapes, respectively. *, 5-isoprenylindole-3-carboxylate β-glycosyl ester. Source data are provided as a Source Data file.

References:

1. Han, Y., et al. Halichoblelide D, a new elaiophylin derivative with potent cytotoxic activity from mangrove-derived *Streptomyces* sp. 219807. *Molecules* **21**, 970 (2016).
2. Nong, X., Wei, X. & Qi, S. Pteridic acids C-G spirocyclic polyketides from the marine-derived *Streptomyces* sp. SCSGAA 0027. *J. Antibiot.* **70**, 1047-1052 (2017).
3. Wu, C., et al. Identification of elaiophylin derivatives from the marine-derived actinomycete *Streptomyces* sp. 7-145 using PCR-bsed screening. *J. Nat. Prod.* **76**, 2153-2157 (2013).
4. Cikosova, M., Blazsek, M., Kubis, M., Gajdosikova, J. & Borosova, G. Biotechnological preparation of the elaiophylin. *Folia Microbiol.* **49**, 731-736 (2004).
5. Yin, M., Jiang, M. X., Ren, Z., Dong, Y. & Lu, T. The complete genome sequence of *Streptomyces autolyticus* CGMCC 0516, the producer of geldanamycin, autolytimycin, reblastatin and elaiophylin. *J. Biotechnol.* **252**, 27-31 (2017).
6. Grabley, S., Hammann, P., Raether, W., Wink, J. & Zeeck, A. Secondary metabolites by chemical screening II. Amycins A and B, two novel niphimycin analogs isolated from a high producer strain of elaiophylin and nigericin. *J. Antibiot.* **43**, 639-647 (1990).
7. Supong, K., et al. Investigation on antimicrobial agents of the terrestrial *Streptomyces* sp. BCC71188. *Appl. Microbiol. Biotechnol.* **101**, 533-543 (2017).
8. Supong, K. & Tanasupawat, S. Secondary metabolites and biological activity of Actinomycetes. *Biotechnology of Microorganisms* 85-106 (2019).
9. Supong, K., et al. Antimicrobial compounds from endophytic *Streptomyces* sp. BCC72023 isolated from rice (*Oryza sativa* L.). *Res. Microbiol.* **167**, 290-298 (2016).
10. Sheng, Y., et al. Identification of elaiophylin skeletal variants from the Indonesian *Streptomyces* sp. ICBB 9297. *J. Nat. Prod.* **78**, 2768-2775 (2015).
11. Arai, M. Azalomycins B and F, two new antibiuotics. II. Properties of azalomycins B and F. *J. Antibiotics* **13**, 51-56 (1960).
12. Lee, S., Ha, S., Hong, Y., Hong, S. & Lee, J. Production of elaiophylin by the strain MCY-846 in a submerged culture. *J. Microbiol. Biotechnol.* **7**, 278-281 (1997).
13. Zhang, Y., et al. Elaiophylin from deep South China Sea-derived *Streptomyces albiflaviniger* SCSIO ZJ28. *Nat. Prod. Res. Dev.* **25**, 185-189 (2013).
14. Buedenbender, L., et al. HSQC-TOCSY fingerprinting-directed discovery of antiplasmodial polyketides from the marine ascidian-derived *Streptomyces* sp. (USC-16018). *Mar. Drugs* **16**, 189 (2018).
15. Komaki, H., Hosoyama, A., Ichikawa, N., Panbangred, W. & Igarashi, Y. Draft genome sequence of *Streptomyces* sp. SPMA113, a prajinamide producer. *Genome Announc.* **4**, e01126-16 (2016).
16. Zhao, G., et al. 16, 17-dihydroxycyclooctatin, a new diterpene from *Streptomyces* sp. LZ35. *Drug Discov. Ther.* **7**, 185-188 (2013).
17. Xu, J., Zhang, X., Huang, F., Li, G. & Leadlay, P. F. Efophylins A and B, two C2-asymmetric macrodiolide immunosuppressants from *Streptomyces malaysiensis*. *J. Nat. Prod.* **84**, 1579-1586 (2021).
18. Guo, Z., Liu, S., Ma, S. & Wang, R. Antibacterial metabolites from the mycelia of the cockchafer-derived *Streptomyces* sp. BCa1. *Chinese Journal of Tropical Crops* **36**, 1307-1311 (2015).
19. Ishibashi, M. Screening study of cancer-related cellular signals from microbial natural products. *J. Antibiot.* **74**, 629-638 (2021).
20. Wang, C., Wang, L., Fan, J., Sun, K. & Zhu, W. Cytotoxic compounds from the deep-sea sediment-derived *Streptomyces malaysiensis* OUCMDZ-2167. *Chinese J. Org. Chem.* **37**, 658-666 (2017).
21. Bown, L. & Bignell, D. R. D. Draft genome sequence of the plant pathogen *Streptomyces*

sp. strain 11-1-2. *Genome Announc.* **5**, (2017).
22. Zhu, J., et al. Complete genome sequence of *Streptomyces malaysiensis* HNM0561, a marine sponge-associated actinomycete producing malaymycin and mccrearamycin E. *Mar. Genom.* **63**, 100947 (2022).
23. Herdini, C., et al. Secondary bioactive metabolite gene clusters identification of anticandida-producing *Streptomyces* sp. GMR22 isolated from Wanagama forest as revealed by genome mining approach. *Indones. J. Pharm.* **28**, 26-33 (2017).
24. Nachtigall, J., et al. Benzoxacystol, a benzoxazine-type enzyme inhibitor from the deep-sea strain *Streptomyces* sp. NTK 935. *J. Antibiot.* **64**, 453-457 (2011).
25. Williams, D., et al. Structures of nahuoic acids B-E produced in culture by a *Streptomyces* sp. isolated from a marine sediment and evidence for the inhibition of the histone methyl transferase SETD8 in human cancer cells by nahuoic acid A. *J. Org. Chem.* **81**, 1324-1332 (2016).
26. He, W., et al. Crossregulation of rapamycin and elaiophylin biosynthesis by RapH in *Streptomyces rapamycinicus*. *Appl. Microbiol. Biotechnol.* **106**, 2147-2159 (2022).
27. Cui, C., Wang, H., Han, B. & Song, Y. Elaiophylins, new cell cycle inhibitors and apoptosis inducers, produced by *Streptomyces pseudoverticillus* (III) structural and NMR studies. *Chinese J. Org. Chem.* **11**, 25-31 (2001).
28. Zhang, H., et al. Secondary metabolites and biosynthetic gene clusters analysis of deep-sea hydrothermal vent-derived *Streptomyces* sp. SCSIO ZS0520. *Mar. Drugs* **20**, (2022).
29. Yang, S., Huang, W., Wang, S. & Wu, J. Extraction, purification and structure elucidation of antibiotic M1 in mycelia of *Streptomyces hygroscopicus* NND-52. *Zhongguo kang sheng su za zhi* **26**, 161-164 (2001).
30. Takesako, K. & Beppu, T. Studies on new antifungal antibiotics, guanidylfungins A and B I. Taxonomy, fermentation, isolation and characterization. *J. Antibiot.* **37**, 1161-1169 (1984).
31. Igarashi, Y., Iida, T., Yoshida, R. & Furumai, T. Pteridic acids A and B, novel plant growth promoters with auxin-like activity from *Streptomyces hygroscopicus* TP-A0451. *J. Antibiot.* **55**, 764-767 (2002).
32. Ilic, S., Konstantinovic, S., Cvijovic, G. & Veljkovic, V. Antibiotic production by *Streptomyces hygroscopicus* CH-7 in medium containing Schiff base complexes. *Hem. Ind.* **73**, 93-101 (2019).
33. Li, S., et al. Rapid identification of elaiophylin from *Streptomyces hygroscopicus* 17997, a geldanamycin producer. *Sheng wu Gong Cheng xue bao* **27**, 1109-1114 (2011).
34. Lima, S., et al. Characterization of the biochemical, physiological, and medicinal properties of *Streptomyces hygroscopicus* ACTMS-9H isolated from the Amazon (Brazil). *Appl. Microbiol. Biotechnol.* **101**, 711-723 (2017).
35. Martín, J., Ramos, A. & Liras, P. Regulation of geldanamycin biosynthesis by cluster-situated transcription factors and the master regulator PhoP. *J. Antibiot.* **8**, 87 (2019).
36. Chung, Y., et al. Comparative genomics reveals a remarkable biosynthetic potential of the *Streptomyces* phylogenetic lineage associated with rugose-ornamented spores. *Msystems* **6**, e00489-21 (2021).
37. Zhou, Y., Li, C., Xu, Y., Wu, W., Xia, W., Huang, X., Huang, D. & Zhou, S. Analysis of complete genome sequence of *Streptomyces solisilvae* HNM0141 of the Streptomyces violaceusniger clade. *Chinese Journal of Tropical Crops* **43**, 455-462 (2022).
38. Yang, H., Zhang, Z., Yan, R., Wang, Y. & Zhu, D. Draft genome sequence of *Streptomyces* sp. strain PRh5, a novel endophytic actinomycete isolated from Dongxiang wild rice root. *Genome Announc.* **2**, e00012-14 (2014).
39. Haltli, B. Elaiophylin Biosynthetic Gene Cluster. *U.S. Patent* 7,595,187 (2009).
40. Klassen, J., Lee, S., Poulsen, M., Beemelmanns, C. & Kim, K. Efomycins K and L from a termite-associated *Streptomyces* sp. M56 and their putative biosynthetic origin. *Front.*

Microbiol. **10**, 1739 (2019).
41. Xin, W., et al. Studies on secondary metabolites of *Acanthopanax senticosus* endophyte *Streptomyces* sp. CWJ-256. *Zhongguo Kang Sheng Su Za Zhi* **42**, 891-895 (2018).
42. Qi, S. Screening of marine actinomycetes producing antimicrobial sustances and study on their secondary metabolites and activities. *Nanchang University* (2020).
43. She, Z., et al. Investigation on the secondary metabolites and activities of four Actinomycetes strains related medicinal plants. *Xinjiang Medical University* (2017).
44. Muller, H., et al. Efomycins a, e and g as Antiinflammatory Agents. *U.S. Patent* 5,185,326A (1993).
45. Nair, M., et al. Gopalamicin, an antifungal macrodiolide produced by soil Actinomycetes. *J. Agric. Food Chem.* **42**, 2308-2310 (1994).
46. Yamada, T., Minoura, K. & Numata, A. Halichoblelide, a potent cytotoxic macrolide from a *Streptomyces* species separated from a marine fish. *Tetrahedron Lett.* **43**, 1721-1724 (2002).
47. Boya P, C., et al. Imaging mass spectrometry and MS/MS molecular networking reveals chemical interactions among cuticular bacteria and pathogenic fungi associated with fungus-growing ants. *Sci. Rep.* **7**, 5604 (2017).
48. Ritzau, M., Heinze, S., Fleck, W., Dahse, H. & Grafe, U. New macrodiolide antibiotics, 11-O-monomethyl- and 11,11 '-O-dimethylelaiophylins, from *Streptomyces* sp. HKI-0113 and HKI-0114. *J. Nat. Prod.* **61**, 1337-1339 (1998).
49. Ivanova, V., Schlegel, R. & Dornberger, K. N '-methylniphimycin, a novel minor congener of niphimycin from *Streptomyces* spec. 57-13. *J. Basic Microbiol.* **38**, 415-419 (1998).
50. Hamedi, J., et al. *Streptomyces iranensis* sp. nov., isolated from soil. *Int. J. Syst. Evol. Microbiol.* **60**, 1504-1509 (2010).
51. Tong, Y., et al. Highly efficient DSB-free base editing for streptomycetes with CRISPR-BEST. *Proc. Natl. Acad. Sci. U. S. A.* **116**, 20366-20375 (2019).
