# Supplementary material for: Streptomyces alleviate abiotic stress in plant by producing pteridic acids
Source: Nat Commun. 2023 Nov 15;14:7398. doi: 10.1038/s41467-023-43177-3 (PMC10652019; doi:10.1038/s41467-023-43177-3)
Supplement: Supplementary file 3 — Description of Additional Supplementary Files [file 41467_2023_43177_MOESM3_ESM.pdf]

## Description of Additional Supplementary Files

File Name: **Supplementary Data 1**
Description: The list of all differentially expressed genes in PH vs. CK and PF vs. CK groups.

File Name: **Supplementary Data 2**
Description: Gene Ontology (GO) and Kyoto Encyclopedia of Genes and Genomes (KEGG) pathway enrichment analysis of differentially expressed genes in PH vs. CK and PF vs. CK groups.

File Name: **Supplementary Data 3**
Description: Differentially expressed transcription factors in PH vs. CK and PF vs. CK groups.
