# Supplementary material for: Streptomyces alleviate abiotic stress in plant by producing pteridic acids
Source: Nat Commun. 2023 Nov 15;14:7398. doi: 10.1038/s41467-023-43177-3 (PMC10652019; doi:10.1038/s41467-023-43177-3)
Supplement: Supplementary file 7 — Reporting Summary [file 41467_2023_43177_MOESM7_ESM.pdf]

nature portfolio

Corresponding author(s): Ling Ding

Last updated by author(s): Oct 21, 2023

Reporting Summary

Nature Portfolio wishes to improve the reproducibility of the work that we publish. This form provides structure for consistency and transparency in reporting. For further information on Nature Portfolio policies, see our Editorial Policies and the Editorial Policy Checklist.

Statistics

For all statistical analyses, confirm that the following items are present in the figure legend, table legend, main text, or Methods section.

n/a	Confirmed	
☐	☒	The exact sample size (*n*) for each experimental group/condition, given as a discrete number and unit of measurement
☐	☒	A statement on whether measurements were taken from distinct samples or whether the same sample was measured repeatedly
☐	☒	The statistical test(s) used AND whether they are one- or two-sided<br>*Only common tests should be described solely by name; describe more complex techniques in the Methods section.*
☒	☐	A description of all covariates tested
☒	☐	A description of any assumptions or corrections, such as tests of normality and adjustment for multiple comparisons
☐	☒	A full description of the statistical parameters including central tendency (e.g. means) or other basic estimates (e.g. regression coefficient) AND variation (e.g. standard deviation) or associated estimates of uncertainty (e.g. confidence intervals)
☐	☒	For null hypothesis testing, the test statistic (e.g. *F*, *t*, *r*) with confidence intervals, effect sizes, degrees of freedom and *P* value noted<br>*Give P values as exact values whenever suitable.*
☒	☐	For Bayesian analysis, information on the choice of priors and Markov chain Monte Carlo settings
☒	☐	For hierarchical and complex designs, identification of the appropriate level for tests and full reporting of outcomes
☒	☐	Estimates of effect sizes (e.g. Cohen's *d*, Pearson's *r*), indicating how they were calculated

Our web collection on statistics for biologists contains articles on many of the points above.

Software and code

Policy information about availability of computer code

Data collection	No software used for data collection
Data analysis	The HR-LC-MS/MS data was processed by MassHunter software (Agilent), GNPS (https://gnps.ucsd.edu/) and MS-Dial version 4.9.2. The antiSMASH version 6.0 was used to predict biosynthetic gene clusters. In mRNA-seq analysis, DESeq2 was used to estimate Differentially Expressed Genes (DEGs) between different treatments. The software g:Profiler and clusterProfiler R package were used in DEGs analysis. BiG-FAM, BiG-SCAPE and CLC Genomics Workbench version 22.0.2 (Qiagen) were employed to conduct comparative genomics analysis. All other softwares used in this study for data analysis were described in the manuscript.

For manuscripts utilizing custom algorithms or software that are central to the research but not yet described in published literature, software must be made available to editors and reviewers. We strongly encourage code deposition in a community repository (e.g. GitHub). See the Nature Portfolio guidelines for submitting code & software for further information.

Data

Policy information about availability of data

All manuscripts must include a data availability statement. This statement should provide the following information, where applicable:

- Accession codes, unique identifiers, or web links for publicly available datasets
- A description of any restrictions on data availability
- For clinical datasets or third party data, please ensure that the statement adheres to our policy

All genome data used in this study were collected from NCBI and the accession numbers were given in Supporting Information file.

Research involving human participants, their data, or biological material

Policy information about studies with human participants or human data. See also policy information about sex, gender (identity/presentation), and sexual orientation and race, ethnicity and racism.

Reporting on sex and gender	Not applicable
Reporting on race, ethnicity, or other socially relevant groupings	Not applicable
Population characteristics	Not applicable
Recruitment	Not applicable
Ethics oversight	Not applicable

Note that full information on the approval of the study protocol must also be provided in the manuscript.

Field-specific reporting

Please select the one below that is the best fit for your research. If you are not sure, read the appropriate sections before making your selection.

☒ Life sciences ☐ Behavioural & social sciences ☐ Ecological, evolutionary & environmental sciences

For a reference copy of the document with all sections, see nature.com/documents/nr-reporting-summary-flat.pdf

Life sciences study design

All studies must disclose on these points even when the disclosure is negative.

Sample size	No statistical methods were used to predetermine sample sizes. The exact number of samples in each experiment was specified in the figure legends. In all cases, sample sizes were adequate as the results were reproducible between different experimental groups.
Data exclusions	No data were intentionally excluded.
Replication	All plant experiments have at least three biological replicates.
Randomization	Plants used for experiments were grown randomly in the same growth chamber.
Blinding	Not applicable

Reporting for specific materials, systems and methods

We require information from authors about some types of materials, experimental systems and methods used in many studies. Here, indicate whether each material, system or method listed is relevant to your study. If you are not sure if a list item applies to your research, read the appropriate section before selecting a response.

Materials & experimental systems

n/a	Involved in the study
☒	☐ Antibodies
☒	☐ Eukaryotic cell lines
☒	☐ Palaeontology and archaeology
☒	☐ Animals and other organisms
☒	☐ Clinical data
☒	☐ Dual use research of concern
☐	☒ Plants

Methods

n/a	Involved in the study
☒	☐ ChIP-seq
☒	☐ Flow cytometry
☒	☐ MRI-based neuroimaging

Dual use research of concern

Policy information about dual use research of concern

Hazards

Could the accidental, deliberate or reckless misuse of agents or technologies generated in the work, or the application of information presented in the manuscript, pose a threat to:

No	Yes
☒	☐ Public health
☒	☐ National security
☒	☐ Crops and/or livestock
☒	☐ Ecosystems
☒	☐ Any other significant area

Experiments of concern

Does the work involve any of these experiments of concern:

No	Yes
☒	☐ Demonstrate how to render a vaccine ineffective
☒	☐ Confer resistance to therapeutically useful antibiotics or antiviral agents
☒	☐ Enhance the virulence of a pathogen or render a nonpathogen virulent
☒	☐ Increase transmissibility of a pathogen
☒	☐ Alter the host range of a pathogen
☒	☐ Enable evasion of diagnostic/detection modalities
☒	☐ Enable the weaponization of a biological agent or toxin
☒	☐ Any other potentially harmful combination of experiments and agents

Plants

Seed stocks	The seeds of Arabidopsis thaliana Col-0 were collected in our lab; The seeds of barley were kindly provided by Thomas Georg Roitsch Lab from the University of Copenhagen; The seeds of Kidney bean and Mung bean were purchased from URTEKRAM and GOLDEN CHEF, respectively.
Novel plant genotypes	Not applicable
Authentication	Not applicable
